# Multiple environmental antigens may trigger autoimmunity in psoriasis through T-cell receptor polyspecificity

**DOI:** 10.3389/fimmu.2024.1374581

**Published:** 2024-03-08

**Authors:** Tatsushi Ishimoto, Yukiyasu Arakawa, Secil Vural, Julia Stöhr, Sigrid Vollmer, Adrian Galinski, Katherina Siewert, Geraldine Rühl, Yuri Poluektov, Marc Delcommenne, Orsolya Horvath, Mengwen He, Burkhard Summer, Ralf Pohl, Rehab Alharbi, Klaus Dornmair, Akiko Arakawa, Jörg C. Prinz

**Affiliations:** ^1^ Department of Dermatology and Allergy, University Hospital, Ludwig-Maximilian-University Munich, Munich, Germany; ^2^ Institute of Clinical Neuroimmunology, Biomedical Center and University Hospital, Ludwig-Maximilian-University Munich, Munich, Germany; ^3^ MBL International, Woburn, MA, United States

**Keywords:** psoriasis, pathogenic T-cell receptor, T-cell receptor polyspecificity, autoimmune response, environmental antigens, diseases triggers, HLA-C*06:02

## Abstract

**Introduction:**

Psoriasis is a T-cell mediated autoimmune skin disease. HLA-C*06:02 is the main psoriasis-specific risk gene. Using a Vα3S1/Vβ13S1 T-cell receptor (TCR) from a lesional psoriatic CD8^+^ T-cell clone we had discovered that, as an underlying pathomechanism, HLA-C*06:02 mediates an autoimmune response against melanocytes in psoriasis, and we had identified an epitope from ADAMTS-like protein 5 (ADAMTSL5) as a melanocyte autoantigen. The conditions activating the psoriatic autoimmune response in genetically predisposed individuals throughout life remain incompletely understood. Here, we aimed to identify environmental antigens that might trigger autoimmunity in psoriasis because of TCR polyspecificity.

**Methods:**

We screened databases with the peptide recognition motif of the Vα3S1/Vβ13S1 TCR for environmental proteins containing peptides activating this TCR. We investigated the immunogenicity of these peptides for psoriasis patients and healthy controls by lymphocyte stimulation experiments and peptide-loaded HLA-C*06:02 tetramers.

**Results:**

We identified peptides from wheat, *Saccharomyces cerevisiae*, microbiota, tobacco, and pathogens that activated both the Vα3S1/Vβ13S1 TCR and CD8^+^ T cells from psoriasis patients. Using fluorescent HLA-C*06:02 tetramers loaded with ADAMTSL5 or wheat peptides, we find that the same CD8^+^ T cells may recognize both autoantigen and environmental antigens. A wheat-free diet could alleviate psoriasis in several patients.

**Discussion:**

Our results show that due to TCR polyspecificity, several environmental antigens corresponding to previously suspected psoriasis risk conditions converge in the reactivity of a pathogenic psoriatic TCR and might thus be able to stimulate the psoriatic autoimmune response against melanocytes. Avoiding the corresponding environmental risk factors could contribute to the management of psoriasis.

## Graphical Abstract

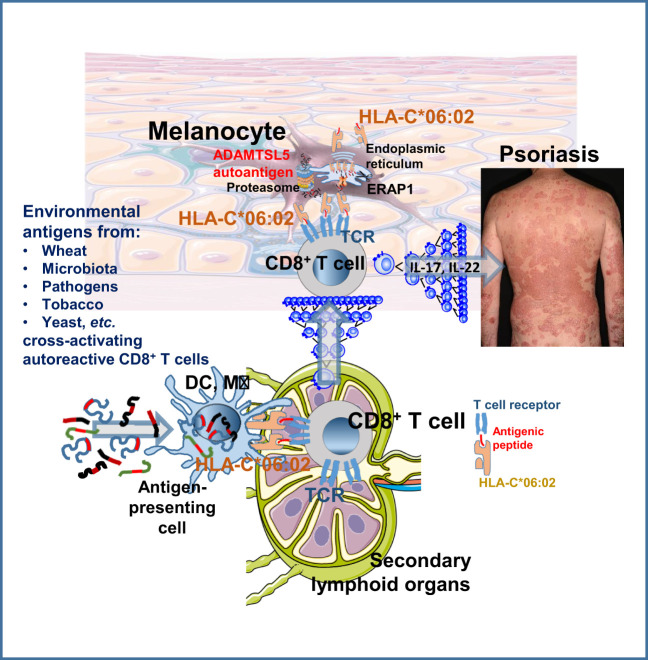

## Introduction

Psoriasis is a common T-cell mediated autoimmune skin disease frequently accompanied by arthritis (1, 2). Within a complex genetic predisposition (3), HLA-C*06:02 is the main psoriasis-specific risk gene (4). Psoriatic skin lesions develop upon epidermal infiltration and activation of CD8^+^ T cells that exhibit marked clonality and conserved TCR patterns, indicating that common disease-specific autoantigens may drive autoimmune T-cell activation in psoriasis (5–9).

Despite the genetic predisposition, psoriasis is not a congenital disease. The typical age of onset in the 2nd and 3rd decade of life (10) and the concordance rate in identical twins, which ranges from 20% to 73% (11, 12), support that environmental, infectious and lifestyle factors may be crucial for initiating autoimmune pathology in psoriasis. Identifying antigenic triggers that convert the genetic predisposition into active autoimmune disease by stimulating the psoriatic autoimmune response against melanocytes could enable causally oriented preventive and therapeutic strategies. However, since the initiating circumstances often remain unclear, treatments for psoriasis are primarily directed at the terminal inflammatory phase (1).

By analyzing the reactivity of a TCR from a lesional psoriatic CD8^+^ T-cell clone rearranging the Vα3S1 and Vβ13S1 variable region genes characteristic of infiltrating lesional CD8^+^ T cells (Vα3S1/Vβ13S1 TCR) (5, 7), we had previously established as an underlying pathomechanism in psoriasis that HLA-C*06:02 may mediate an autoimmune response against melanocytes, and we had identified an epitope from ADAMTS-like protein 5 (ADAMTSL5) as a melanocyte autoantigen presented by HLA-C*06:02 (13, 14). Extensive immunohistological studies further confirmed that the CD8^+^ T cells in lesional psoriatic epidermis indeed react against melanocytes, as indicated by the Vα3S1/Vβ13S1 TCR (13). Unlike the melanocyte-depleting cytotoxic autoimmune response causing vitiligo, CD8^+^ T cells activated against melanocytes in psoriasis cause chronic inflammation through the production of IL-17 and IL-22 (15–19).

A suspected mechanism for self-reactive T cells to evade peripheral tolerance mechanisms is through the antigen recognition mode of TCRs. TCRs are polyspecific and ligated by antigenic peptides sharing a certain amino acid motif (20, 21). It is defined by two or three amino acids anchoring the peptides in the discrete pockets of the peptide binding groove of the cognate HLA molecule, and one or two TCR-specific contact residues, while tolerating a broad amino acid diversity at other positions of the antigenic peptides. Therefore, it was postulated that due to the extensive TCR cross-reactivity environmental antigens may induce autoimmune diseases by stimulating potentially autoreactive T cells (22). Accordingly, in ankylosing spondylitis (AS), which is part of the psoriatic arthritis spectrum, TCRs of clonally expanded CD8^+^ T cells from synovial fluid or uveitis were found to recognize both microbial and self-peptides presented by the AS-specific risk allele HLA-B*27 (23).

To identify environmental peptides that ligate the Vα3S1/Vβ13S1 TCR and thus might stimulate the autoimmune response against melanocytes in psoriasis, we searched protein databases for proteins containing the peptide recognition motif of this TCR. We identified several environmental and microbial antigens that correspond to previously suspected psoriasis risk conditions and stimulated the Vα3S1/Vβ13S1 TCR and CD8^+^ T cells of psoriasis patients. Thus, our results directly support in patients that due to the polyspecificity of TCRs, different environmental antigens may stimulate autoreactive T cells and promote autoimmunity in psoriasis.

## Materials and methods

### Study design

The study was designed to identify environmental antigens that can trigger a psoriatic autoimmune response due to TCR polyspecificity. We performed a sequential approach: 1. Precise characterization of the peptide recognition motif of an autoreactive Vα3S1/Vβ13S1 TCR from a lesional psoriatic CD8^+^ T cell clone specific for a melanocytic psoriatic autoantigen. 2. Use of the peptide recognition motif to search databases for environmental antigens that activate the autoreactive Vα3S1/Vβ13S1 TCR. 3. Verification of the immunogenicity of candidate antigens for CD8^+^ T cells from psoriasis patients. 4. Investigation of whether the same CD8^+^ T cells can recognise both autoantigen and environmental peptides using peptide-loaded HLA-C*06:02 tetramers. 5. Follow-up of the effect of exposure prophylaxis of certain environmental antigens (wheat) on the course of psoriasis.

### Patients providing blood samples

41 Patients with type 1 chronic plaque psoriasis (early onset, positive family history and/or HLA-C*06:02 (10)) were randomly recruited from the psoriasis outpatient clinic of the Department of Dermatology and Allergy, University Hospital, Ludwig-Maximilian-University, to represent the normal patient distribution. 12 healthy subjects without a personal or family history of psoriasis or psoriatic arthritis served as controls selected to approximately match age. Median age of Pso was 47.6 y, STDEV 14.43 y; median age of HC was 44.9 y, STDEV 12.75 y; *P* = 0.0840. Patients and healthy controls participated voluntarily and gave their written informed consent.

### Patients undergoing a gluten-free diet

Several patients with inadequate response to their current therapy and missing therapeutic alternatives due to contraindications to other drugs (pre-existing malignancies, chronic infections), lack of response to or side effects of previously taken drugs, or concern about these, opted for a GfD because of our experimental findings. We followed the course of psoriasis at routine patient visits by means of the Psoriasis Area and Severity Index (PASI) and the Physicians Global Assessment (PGA), the standard of care parameters for measuring psoriasis activity, for six months or longer. Patients who showed a substantial improvement in terms of a PASI 75 score or better within three months continued with GfD to maintain the benefit. Dietary errors occurred during holiday periods abroad (Paris, Rome) when patients were unable to adhere to GfD for language reasons. Patient data are summarized in **Table 1**.

### Motif-based searches for naturally occurring peptide ligands of the Vα3S1/Vβ13S1TCR

Sequence logos of previously identified mimotopes and self-peptide ligands of the Vα3S1/Vβ13S1 TCR were created at WebLogo (https://weblogo.berkeley.edu/logo.cgi). Emboss prophecy (http://emboss.sourceforge.net/apps/release/6.6/emboss/apps/prophecy.html) was used to create search motifs from positively tested antigenic peptides. The main octamer search motifs were RXXRXXRL, RXXNXRRL, RXXRXRRL, RXXNXLRL, RXXRXLRL, RXXQXLRL, RXXRXLRM, and RXXRXRRM. Unrestricted searches at the UniProtKB/Swiss-Prot databases were carried out using the Expasy ScanProsite tool and the Protein BLAST Basic Local Alignment Search Tool (https://prosite.expasy.org/scanprosite/scanprosite_doc.html, https://blast.ncbi.nlm.nih.gov/Blast.cgi). The list of results was manually curated for amino acids at positions 3-4 and 6 seen in the heat maps and sequence logos of previously identified mimotopes and stimulatory peptides (**Supplementary Figure S1A**, **Supplementary Table S1**). In nonamers, amino acids at P1 were supplemented according to the natural protein sequence. Binding to HLA-C*06:02 was verified using the NetMHCpan - 4.1 server (https://services.healthtech.dtu.dk/service.php?NetMHCpan-4.1).

### Generation of plasmid-encoded peptides for Vα3S1/Vβ13S1-TCR hybridoma stimulation

For expression of short candidate peptides, forward and reverse oligonucleotides (Biomers) carrying a 5’-CACCATG overhang and a stop codon in 3’-position of the target sequence (**Supplementary Table S2**) were annealed and ligated into pcDNA3.1D/V5-His-TOPO using pcDNA3.1 Directional TOPO Expression Kit (Invitrogen, Carlsbad, USA) according to manufacturer´s instructions as described (13, 14).

### Culture conditions and stimulation of the CD8^+^ Vα3S1/Vβ13S1-TCR reporter T-hybridoma cell line

Generation and culture conditions of the Vα3S1/Vβ13S1-TCR CD8^+^ reporter T hybridoma from the paired αβ-TCR chains rearranging the Vα3S1 and the Vβ13S1 gene segments of a lesional psoriatic CD8^+^ T-cell clone have been described (7, 13). RPMI 1640 culture medium contained 10% FCS, 100 units/ml penicillin, 100 µg/ml streptomycin, 1 mM sodium pyruvate, 1x MEM non-essential amino acids and 10 µg/ml ciprofloxacin, supplemented with selection antibiotics G418 (1.5 mg/ml), hygromycin (300 µg/ml, both Genaxxon), puromycin (1 µg/ml, Sigma) and blasticidin (3 µg/ml, PAA). For co-culture experiments, TCR hybridoma cells were pelleted and resuspended in hybridoma growth medium without selection antibiotics before addition to antigen-presenting cells.

To determine the antigenicity of peptide antigens for the Vα3S1/Vβ13S1-TCR hybridoma, COS-7 cells were cotransfected with HLA-C*06:02- and peptide-encoding plasmids (250 ng each) using FuGENE HD reagent (Promega). After 24 h, medium was replaced with fresh medium containing Vα3S1/Vβ13S1-TCR hybridoma cells as described (13, 14). After 24 h of co-culture, the degree of hybridoma activation was determined on a BD FACSCanto Flow Cytometry System with respect to the percentage of sGFP^+^ hybridoma cells. For evaluation of TCR stimulation, Vα3S1/Vβ13S1-TCR hybridoma cells were cultured in culture plates either untreated or pre-coated with CD3 antibody (eBioscience #14-0032-82, 17A2, 2 µg/ml in PBS). The percentage of hybridoma cells induced to express sGFP directly correlates with the degree of TCR ligation and was therefore used to quantify TCR stimulation (14). The maximal possible yield of 50% to 70% of anti-CD3-stimulated sGFP-positive hybridoma cells reflects their maximum activatability also observed in former experiments that had established this technology (24).

### Stimulation of PBMC

Peripheral blood mononuclear cells (PBMC) were separated from heparinized venous blood using Ficoll density gradient centrifugation (Biocoll, Biochrom). They were seeded in 96 well flat bottom plates at 2x10^5^ cells/well and cultured in RPMI 1640 medium supplemented with 10% human AB serum. Highly purified synthetic peptides (> 95%, Thermo Fisher) corresponding to the plasmid-encoded environmental antigens stimulating the Vα3S1/Vβ13S1 TCR were used for stimulation at a final concentration of 10 µg/ml. An unrelated peptide without a human protein correlate previously isolated from HLA-C*06:02 of lymphoblastoid B cells (25), VRHDGGNVL, here named “FALK” peptide, served as a comparison. HLA-C*06:02 typing was performed by PCR restriction fragment length polymorphism analysis as described previously (26), or determined by sequence-based typing at the Laboratory for Immunogenetics and Molecular Diagnostics, University Hospital, Ludwig-Maximilian-University of Munich.

### Measurement of stimulation-induced [^3^H]-thymidine incorporation of PBMC and induction of CD137 expression on CD8^+^ T cells

To measure stimulation-induced proliferation, triplicates of PBMC cells were pulsed overnight on day 5 of stimulation with 24,500 bq/well [^3^H]-thymidine (Hartmann Analytic (Braunschweig, Germany), and proliferation was assessed according to the incorporated radioactivity. Harvesting and measurement of radioactive decay (counts per minute) were done using an automatic filter counting system (Inotech Biosystems, Derwood, USA).

Peptide-induced activation of CD8^+^ T cells was assessed based on the induction of CD137 expression (27). PBMC were incubated with synthetic peptides and harvested after 24 hours. Cells were stained with CD-137 FITC/PE-Cy7 (Clone 4B4-1, eBioscience Cat. Nr. 11-1379-42, BioLegend, Cat.Nr. 309818) and CD8 perCP/Cy5.5 SK1 (BioLegend, Cat.Nr. 344710) for 30 min on ice. For flow cytometry analysis of CD137 expression, lymphocytes were gated using FSC and SSC. Cells gated for CD8 expression were analyzed for the expression of CD137. Isotype IgG controls and unstimulated cells were included in each experiment. At least 1x10^5^ cells per sample were analyzed with a FACSCanto II (Becton Dickinson). FlowJo software v10.7.2 (BD Biosciences USA) was used to analyze the results. A representative analysis is given in **Supplementary Figure S3**.

Peptide-induced activation was expressed as stimulation index (SI), which is the median cpm of PBMC triplicates or percentage of CD137^+^ CD8^+^ T cells of stimulated cells relative to the mean percentage of unstimulated control cells.

### Determination of Gliadin antibodies

Gliadin antibodies were determined in undiluted serum samples by the Alegria® system, a quantitative automated indirect enzyme-linked immune reaction (ORGENTEC Diagnostika GmbH, Mainz, Germany) according to the manufacturer’s instructions. If samples were positive for IgG and IgA antibodies against deamidated gliadin peptide (DGP) epitopes, anti-DGP IgA was determined in a second step. Antibody titers ≥10 U/ml are considered elevated in both tests (https://products.orgentec.com/pdfs/ifu/ORG%20251S_IFU_EN_QM113115_2018-01-02_4.pdf).

### Generation of HLA-C*06:2 tetramers

HLA-C*06:02 tetramers were generated essentially as described with a few minor modifications (28). A DNA construct (IDT) encoding the extracellular domain of HLA-C*06:02, with valine replacing alanine at codon 245 (29) and fused with a sequence encoding for the BirA biotinylation motif was inserted in a bacterial expression vector. The HLA-C*06:02 heavy chain and β_2_-microglobulin (β_2_m) were produced separately in *E. coli*. Bacteria were pelleted by centrifugation and lysed in a buffer containing lysozyme and Triton X-100. After several washes purified inclusion bodies were obtained and solubilized in a urea containing buffer. Inclusion bodies of the HLA-C*06:02 heavy chain and β_2_m were mixed with synthetic nonamer peptides for ADAMTSL5 or wheat-1 (LRMRRCRRM) or wheat-2 (VRAGRVLRV, see **Supplementary Table S2**) in a folding buffer containing a mix of reduced and oxidized glutathione. Folding reactions took place at 4°C in the dark for 3-5 days. The folded complexes (or MHC monomers) were concentrated, dialyzed and biotinylated in a solution containing D-biotin and ATP to which the BirA enzyme (Avidity) was added. Biotinylated monomers were dialyzed against an NaCl 150 mM, Tris 10 mM pH 8.0 buffer and purified on a monomeric streptavidin affinity column (Thermofisher) by elution with D-biotin. The biotinylated complexes were dialyzed against NaCl 150 mM, Tris 10 mM pH 8.0, concentrated and tested by HPLC on a gel filtration column (Cytiva) for determining monomer purity and percentage of free β2m. Monomers were tetramerized with streptavidin conjugated to phycoerythrin or Brilliant Violet 421 at a 4/1 ratio (28). The HLA-C*06:02 tetramers were produced by MBL International, Woburn, MA, and will become commercially available.

### Tetramer staining, gating strategy and flow cytometry analysis

Freshly isolated peripheral blood mononuclear cells (PBMC) (1x10^6^ cells/ml) were resuspended in RPMI 1640 medium supplemented with 1% PenStrep, 10 µg/ml ciprofloxacin and 10% FCS. Cells were incubated with 50 nM dasatinib (Santa Cruz Biotechnology, TX, USA), 50 U/ml IL-2 and 25 mM glucose at 37°C for 30 min (30). Either PE or BV421 labelled HLA-C*06:02 tetramers or both were added at a concentration of 10 µg/ml each and incubated at 37°C for 25 min, and then stained with the following antibodies at 37°C for 30 min: Anti-human-CD3-FITC (Clone SK7, Invitrogen); Anti-human-CD8-PerCp/Cyanine5.5 (Clone: SK1, Biolegend); Anti-human-CD56-PE-Cy7 (Clone: B159, BD Pharmingen). Cells were washed and resuspended with FACS Buffer (PBS with 1% FBS) and then stained with 4’, 6-diamidino-2-phenylindole (DAPI, 0.33 µM) for 5 min at room temperature. All samples were acquired on BD LSRFortessa (BD Biosciences). Data were analyzed using FlowJo software v10.7.2 (FlowJo LLC). Antigen-specific TCR binding of the HLA-C*06:02 tetramers was verified by tetramer staining of the Vα3S1/Vβ13S1 TCR hybridoma and two TCR hybridomas expressing TCRs of unknown specificity (**Figure 4**). The gating strategy that resulted in cell populations consisting of Live/CD3^+^/CD8^+^/CD56^neg^ HLA-C*06:02 tetramer^+^ cells is shown in **Supplementary Figure S4**. Lymphocytes were determined by forward (FSC) and side scatter (SSC) gating. To discriminate live from dead cells, the DAPI negative population was gated. Live cells were gated for the CD56-negative population to minimize the impact of Killer cell Ig-like receptors (KIR) of natural killer cells on tetramer binding. Next, live/CD56^neg^ were gated on the CD3^+^CD8^+^ double-positive cells. FALK-HLA-C*06:02 tetramer was used for comparison to determine the cut-off between background and positive staining of ADAMTSL5-HLA-C*06:02 tetramer^+^ cells. Flow cytometry analysis of PBMC stained with the HLA-C*06:02 tetramers and antibodies specific for CD3, CD56 and the anti-human-Killer-cell immunoglobulin-like receptor (KIR)/CD158, KIR2DL1/2DS1, had excluded binding of the HLA-C*06:02 tetramers to CD3^+^ T cells through KIRs.

### Statistical analysis

Statistical analyses of the data given in **Figures 3**, **4** and **Supplementary Figure S2** were performed using GraphPad Prism software (ver. 8) and further confirmed by XLSTAT ver2014.5. Microsoft Excel was used to store data and to run XLSTAT. For each value of multiple groups, non-parametric Kruskal-Wallis H test was used for multiple comparisons, and Bonferroni correction was applied (*P <*0.0001, for [^3^H]-thymidine incorporation or CD137 expression, HC vs. PV). Two-group comparison was performed using a non-parametric two-sided Mann-Whitney U test for unpaired continuous variables. Two-tailed *P* < 0.05 was considered statistically significant. Age groups of patients and healthy controls were compared using Fisher’s exact test. In principle, no data were excluded from analyses. Some stimulation experiments were excluded when the positive control samples with CD3 antibody stimulation from the same samples had failed or seeding cell numbers were inappropriately adjusted. Investigators were not blinded for samples except measurement of [^3^H]-thymidine incorporation.

## Results

### The peptide motif ligating the ADAMTSL5-specific psoriatic Vα3S1/Vβ13S1 TCR is composed of five amino acids

To identify antigenic psoriasis triggers the HLA-C06:02-restricted psoriatic Vα3S1/Vβ13S1 TCR was expressed in a reporter mouse hybridoma cell line which produces super green fluorescent protein (sGFP) upon TCR ligation (24). We employed HLA-C06:02-cotransfected COS-7 cells to present plasmid-encoded candidate peptides in co-culture experiments and analyzed TCR activation by measuring the percentage of sGFP^+^ hybridoma cells that reflects the degree of TCR stimulation (14).

**Figure 1:**
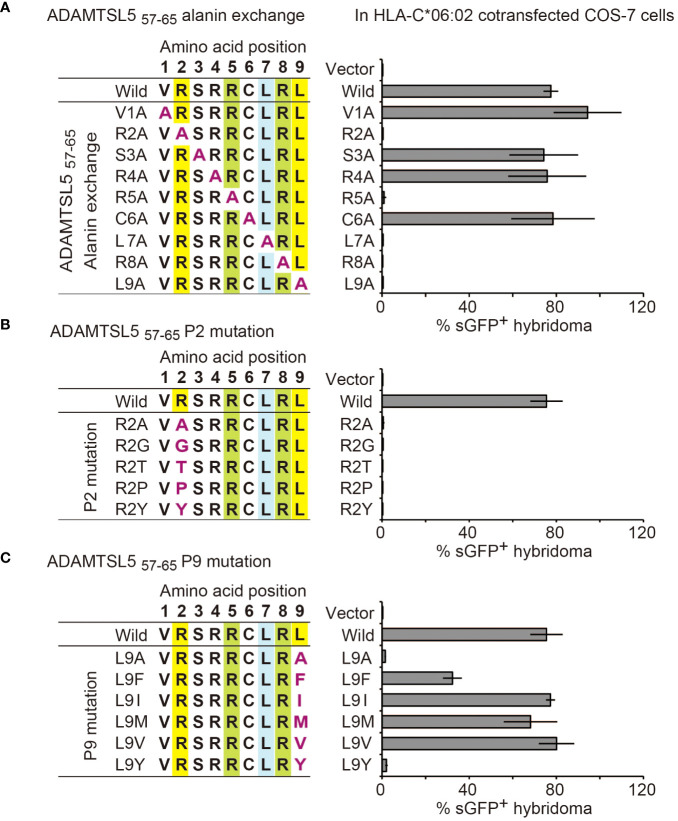
Mutagenesis determines the recognition motif of the ADAMTSL5-specific psoriatic Vα3S1/Vβ13S1 TCR. Relevance of individual amino acid residues of the ADAMTSL5 nonamer peptide for stimulation of the Vα3S1/Vβ13S1-TCR hybridoma as determined by **(A)** alanine scanning mutagenesis or exchange against other HLA-C*06:02 anchor residues at **(B)** P2 or **(C)** P9. Mutated peptides were cloned into plasmids, cotransfected with HLA-C*06:02 in COS-7 cells and used to stimulate the Vα3S1/Vβ13S1 TCR hybridoma in co-culture experiments. Percentages of sGFP^+^ hybridoma cells were assessed by flow cytometry after 24h and normalized to CD3 stimulation data in the same experiment. Data are summarized from duplicates **(A)** or **(B, C)** triplicates of two independent experiments and shown as mean ± SEM.

We first precisely characterized the peptide recognition motif of the Vα3S1/Vβ13S1 TCR. Alanine scanning mutagenesis (31) revealed that exchange of the amino acids at positions P1, P3, P4, and P6 against Ala had no effect on the antigenicity of the ADAMTSL5 nonamer (**Figure 1A**). Ala mutations of the amino acids at P 2, 7, or 9, which anchor the peptide in the HLA-C*06:02 binding groove (32–34), or of the amino acids at P5 or P8, which mediate contact of the ADAMTSL5 peptide with the Vα3S1/Vβ13S1 TCR (33), completely abolished the ability to stimulate the Vα3S1/Vβ13S1 TCR. The antigenicity of the ADAMTSL5 required Arg as HLA-C*06:02 anchor at P2, because it was lost upon exchange against other HLA-C06:02 anchor amino acids at this position (Gly, Thr, Pro, or Tyr) (32–34) (**Figure 1B**), while it tolerated a replacement against other HLA-C*06:02 anchor amino acids at P9 (Phe, Ile, Met, or Val, **Figure 1C**).

We integrated the mutagenesis data and previously identified mimotopes and self-peptide ligands of the Vα3S1/Vβ13S1 TCR (13) into heat maps of amino acid preferences and sequence logos (**Supplementary Table S1**, **Supplementary Figure S1A**). This confirmed that the Vα3S1/Vβ13S1 TCR recognition motif required Arg as HLA-C*06:02 anchor residue at P2 and as TCR contact residue at P8, whereas other positions of the motif showed some variations. P5 was occupied either by Arg, Asn or Gln, while P7 could accommodate Arg or Leu, and P9 was occupied either by Leu, Met, Val, Phe or Ile. All other positions showed greater amino acid diversity. Accordingly, the recognition motif of the Vα3S1/Vβ13S1 TCR is based on the five amino acid positions P2, P5, and P7-P9. It determines the cross-reactivity between different peptides exhibiting this motif. In octamers, the NH_2_-terminus corresponds to P2.

### Motif-based search identifies multiple environmental peptide ligands of the pathogenic psoriatic Vα3S1/Vβ13S1 TCR

We searched the NCBI and UniProt protein databases with the aggregate data of the Vα3S1/Vβ13S1-TCR recognition motif for environmental proteins containing homologous amino acid sequences that may elicit a cross-reactive autoimmune response against the melanocyte autoantigen. For this, we variably combined the fixed amino acids at P2 and P8 in an octamer search matrix with the other amino acids of the TCR recognition motif. For respective nonamers, P1 was completed according to the natural protein sequence.

**Figure 2:**
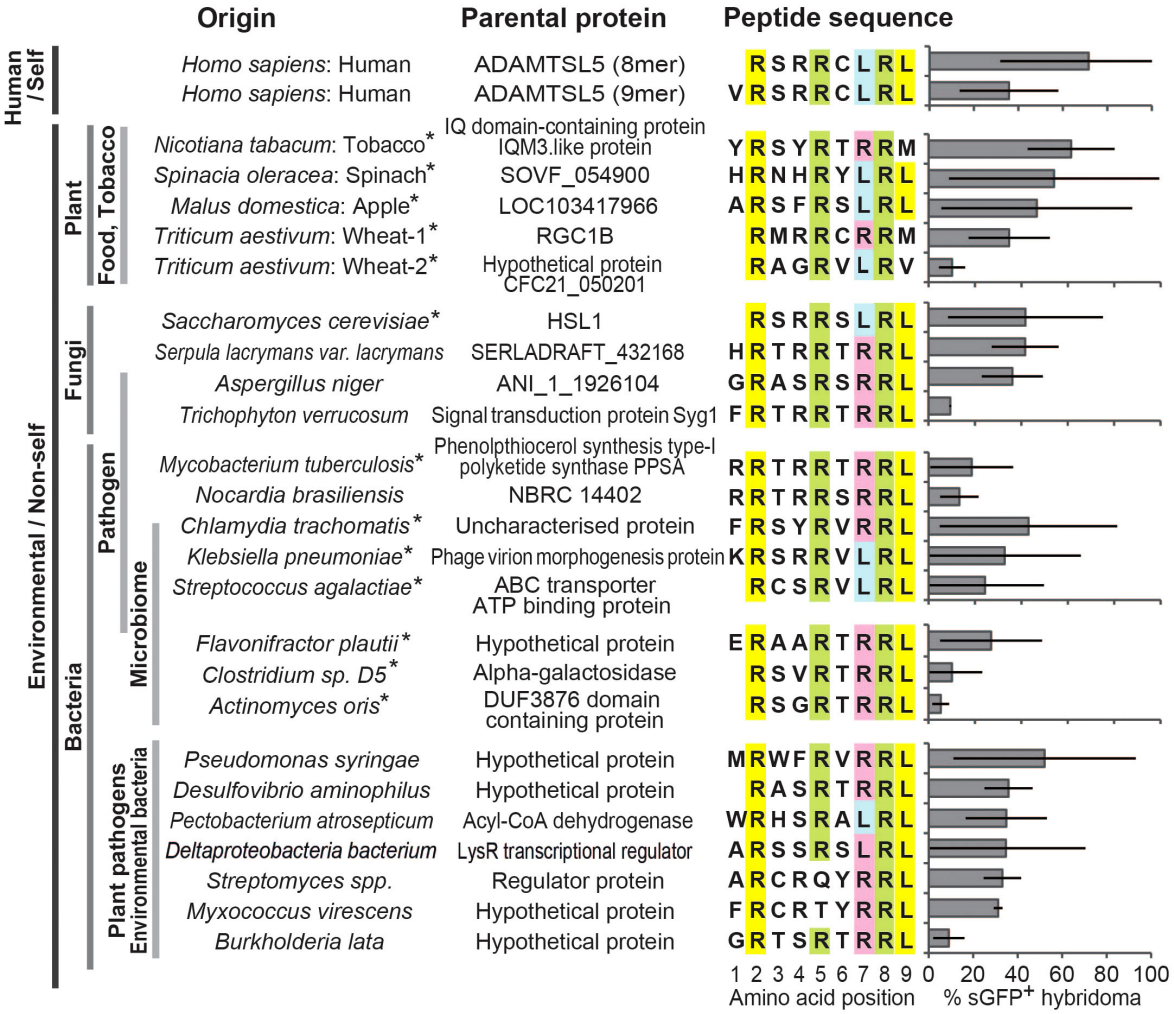
Homology search with the TCR recognition motif identifies various environmental peptide ligands of the ADAMTSL5-specific Vα3S1/Vβ13S1 TCR. Response to stimulation of the Vα3S1/Vβ13S1-TCR hybridoma by co-culture with COS-7 cells cotransfected with HLA-C*06:02 and plasmids encoding the ADAMTSL5 epitope or various environmental candidate antigens. Induction of sGFP was determined by flow cytometry analysis after 24h. Results are given as mean relative to CD3 stimulation, bars represent SD. Data are from at least 3 individual analyses or duplicates from two or more independent experiments. Anchor residues for HLA-C*06:02 at P2 and P9 are highlighted in yellow, the auxiliary anchor at position 7 in blue or red and the TCR contact residues of the Vα3S1/Vβ13S1 TCR recognition motif at positions 5 and 8 in green. Asterisks indicate peptides used to stimulate PBMC.

After verifying high affinity binding to HLA-C*06:02 by the NetMHCPan 4.1 server (https://services.healthtech.dtu.dk/service.php?NetMHCpan-4.1), 50 different environmental peptides were selected from the search results (**Supplementary Table S2**) and used to stimulate the Vα3S1/Vβ13S1 TCR as plasmid-encoded octamers or nonamers. 24 of them stimulated the Vα3S1/Vβ13S1 TCR hybridoma (**Figure 2**). They were derived from various sources that included pathogenic bacteria, fungi, yeasts, skin or mucosal microbiota, and crops.

Thus, the TCR recognition motif had allowed us to identify diverse exogenous peptide ligands of the autoreactive psoriatic Vα3S1/Vβ13S1 TCR. Stimulation with octamer and nonamer peptides from ADAMTSL5, wheat and *Flavonifractor plautii* revealed that the amino acid at P1 is of less importance for the antigenicity, as both the octamers lacking P1 and the nonamers stimulated the Vα3S1/Vβ13S1 TCR, as recently reported (14) (**Supplementary Figure S2**). Sequence comparison between stimulatory and nonstimulatory ligands furthermore showed that the TCR recognition motif alone did not guarantee TCR ligation, as it was also present in non-stimulatory peptides (**Supplementary Figures S1B, C**). In particular, Arg at P6 seemed to prevent activation of the Vα3S1/Vβ13S1 TCR. This indicates that certain amino acids at otherwise neutral peptide positions may alter the contact interface for the Vα3S1/Vβ13S1 TCR.

### Environmental peptides stimulating the autoreactive Vα3S1/Vβ13S1 TCR are particularly immunogenic for CD8^+^ T cells from psoriasis patients

Exogenous peptides stimulating the autoreactive ADAMTSL5-specific Vα3S1/Vβ13S1 TCR are potential environmental triggers of the psoriatic autoimmune response. To assess their immunogenicity directly in patients, we stimulated peripheral blood mononuclear cells (PBMC) from patients with chronic plaque psoriasis or healthy controls with corresponding synthetic peptides. We focused on peptides originating from conditions directly related to the human environment in terms of nutritional and consumer products, infections, and the microbiota. Since the psoriatic autoimmune response is restricted by HLA-C*06:02 (13) and mediated by CD8^+^ T cells (8), we analyzed the antigen-induced T-cell proliferation of PBMC by [^3^H]-thymidine in combination with activation-induced CD137 expression on CD8^+^ T cells (**Supplementary Figure S3**), which are the actual pathogenic T cells in psoriasis (8). CD137 is a member of the TNFR-family. It is induced by functional stimulation on human CD8^+^ T-cells and is a reliable marker for their antigen-specific activation (27). A peptide of unknown origin with the sequence VRHDGGNVL, here referred to as the “FALK” peptide, which is known to bind to HLA-C*06:02 as it was formerly isolated from an HLA-C*06:02^+^ lymphoblastic B cell line (25), served as a reference unrelated to psoriasis pathogenesis.

**Figure 3:**
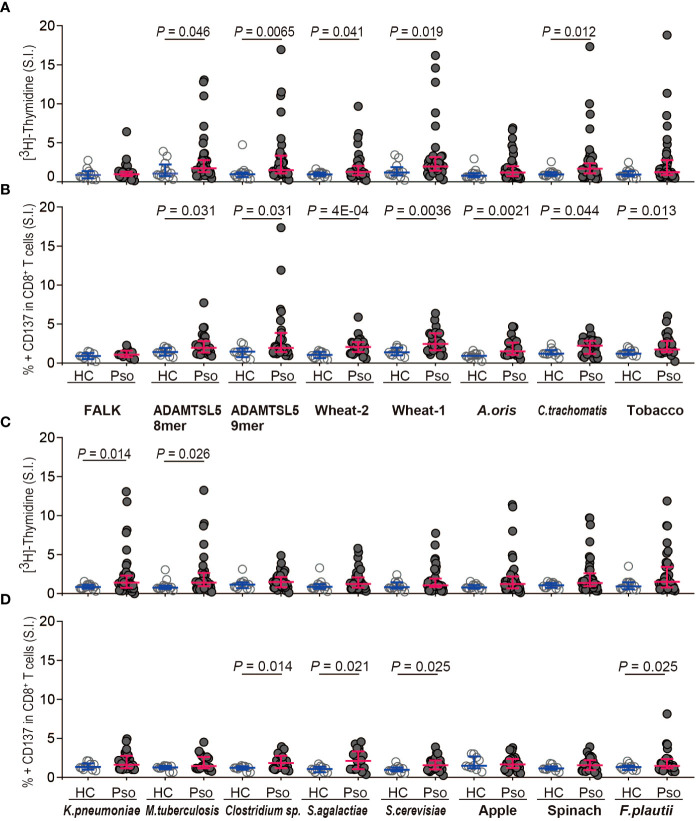
Environmental Vα3S1/Vβ13S1 TCR ligands preferentially activate PBMC and CD8^+^ T cells from psoriasis patients. **(A, C)** [^3^H]-thymidine incorporation by PBMC from healthy controls (HC, *n* = 12) and psoriasis patients (Pso, *n* = 41) stimulated with synthetic peptides shown in **Figure 2** for 5d. The stimulatory response is expressed as stimulation index that represents the fold-increase over the control sample cultured without peptides. FALK peptide served as disease-unrelated control. Each dot represents one individual. Results are given as the median of cpm of triplicates for each sample and compared between groups by non-parametric two-sided Mann-Whitney U test. Bars indicate median and interquartile values. **(B, D)** Activation of CD8^+^ T cells by stimulation with synthetic peptides for 24h as determined by the induction of CD137 expression on CD8^+^ T cells from HC (*n* = 10) and Pso (*n* = 28). Results are given as stimulation index that is calculated from the mean percentage of CD137^+^ CD8^+^ T cells of duplicates. Two-group comparison was performed using Mann-Whitney U test for unpaired continuous variables. *P-*values < 0.05 are indicated.

Measurement of peptide-induced [^3^H]-thymidine incorporation showed that most environmental antigens had higher immunogenicity for psoriasis patients than for healthy subjects (**Figures 3A, C**). Besides the ADAMTSL5 octamer and nonamer, the difference was statistically significant for peptides from wheat (*Triticum aestivum*, wheat-1: RCG1B, wheat-2: Hypothetical protein CFC21_050201), *Chlamydia trachomatis*, *Klebsiella pneumoniae*, and *Mycobacterium tuberculosis*. Analysis of CD137 expression (**Figures 3B, D**) revealed that several peptides induced a significantly higher CD137 expression on CD8^+^ T cells from psoriasis patients than from healthy controls. In addition to ADAMTSL5, they included peptides from wheat, *Actinomyces oris*, *C. trachomatis*, the IQM3-like isoform from *Nicotiana tabacum* (tobacco), *Saccharomyces cerevisiae* as well as *Clostridium* sp. and *Streptococcus agalactiae*, both of which belong to the microbiota. The particular immunogenicity of ADAMTSL5 and wheat peptides for psoriasis patients is highlighted by the coincidence of significantly increased stimulation-induced [^3^H]-thymidine incorporation and CD137 expression on CD8^+^ T cells (**Figures 3A, B**).

### CD8^+^ T cells may recognize both the autoantigen and wheat peptides

To investigate whether, according to the polyspecificity of the Vα3S1/Vβ13S1 TCR, the same CD8^+^ T cells in psoriasis patients can recognize both the ADAMTSL5 peptide and environmental antigens, we employed fluorescent HLA-C*06:02 tetramers loaded either with the ADAMTSL5 or the wheat peptides to stain CD8^+^ T cells from HLA-C*06:02^+^ psoriasis patients and healthy individuals.

**Figure 4:**
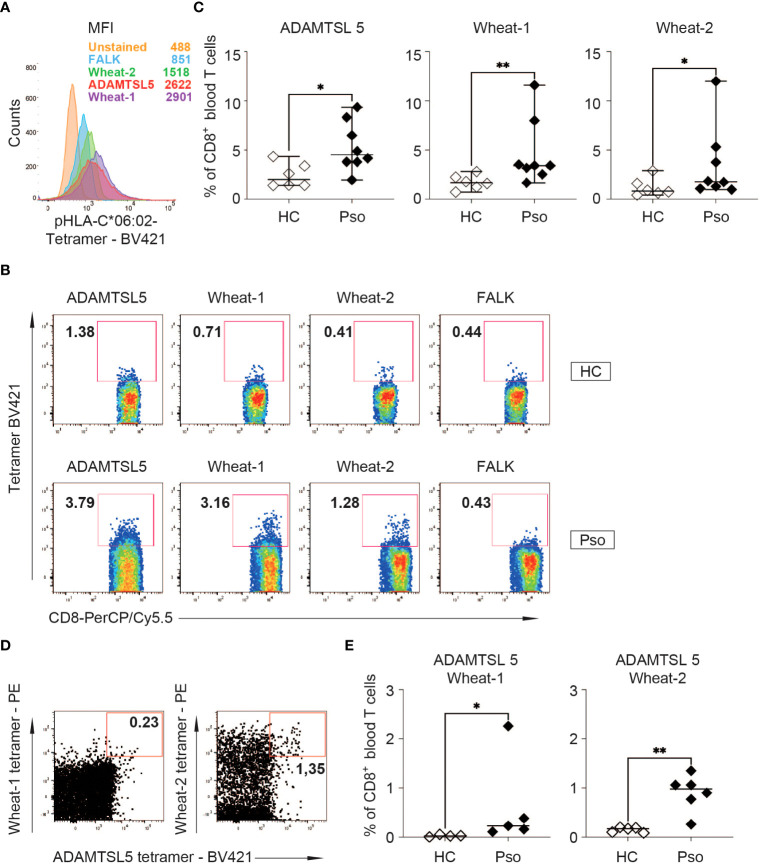
Peptide-HLA-C*06:02 tetramer staining of PBMC shows an increased frequency of ADAMTSL5 and wheat-specific CD8^+^ T cells in psoriasis patients. **(A)** Binding to the Vα3S1/Vβ13S1 TCR hybridoma and mean fluorescence staining intensity (MFI) of Brilliant Violet 421 (BV421)-labelled ADAMTSL5, wheat-1, wheat-2 and FALK peptide-HLA-C*06:02 tetramers. According to CD3 antibody staining, approximately 60% of the hybridoma cells express the Vα3S1/Vβ13S1 TCR. **(B)** Representative staining of PBMCs from a HC and a psoriasis patient with the BV421-labelled ADAMTSL5, wheat-1, wheat-2 and FALK peptide-HLA-C*06:02-tetramers. **(C)** Frequency of CD8^+^ T cells binding ADAMTSL5 or wheat peptide-HLA-C*06:02 tetramers in HLA-C*06:02^+^ healthy controls (HC, *n* = 6) or psoriasis patients (Pso, *n* = 8). **(D, E)** Representative staining of CD8^+^ T cells from a psoriasis patient and frequency of CD8^+^ T cells double-stained with BV421-labelled ADAMTSL5-HLA-C*06:02 tetramers and PE-labelled wheat-1-HLA-C*06:02 (HC: *n* =4; Pso: *n* = 5) or wheat-2-HLA-C*06:02 tetramers (HC: *n* = 5; Pso: *n* = 6). The data in **(C, E)** represent mean values of duplicates. Differences in Live/CD3^+^CD8^+^ tetramer single^+^ or double^+^ cell frequency between HC and Pso **(C, E)** were assessed by Mann-Whitney U test. *P*-values < 0.05 are indicated. * *P* 0.05; ** *P* 0.01.

The binding specificity of the peptide-loaded tetramers was established by staining of the Vα3S1/Vβ13S1-TCR hybridoma with ADAMTSL5 or wheat peptide-loaded but not FALK peptide-loaded HLA-C*06:02 tetramers, which served as disease-unrelated control (**Figure 4A**). None of these peptide-loaded HLA-C*06:02 tetramers bound to hybridomas carrying unrelated TCRs (data not shown). Strict gating of the flow cytometry analyses on CD3^+^ CD8^+^ T cells further excluded that staining results were confounded by tetramer-binding to CD56^+^ natural killer cells or KIR2DL1/2DS1 receptors.

When PBMC were examined, the ADAMTSL5- and wheat-peptide loaded HLA-C*06:02 tetramers stained significantly more CD8^+^ T cells of HLA-C*06:02^+^ psoriasis patients than of HLA-C*06:02^+^ healthy subjects (**Figures 4A–C**). Simultaneous staining with the ADAMTSL5- and wheat-peptide loaded HLA-C*06:02 tetramers showed that a proportion of CD8^+^ T cells can recognize both the autoantigen and environmental peptides. Moreover, significantly more CD8^+^ T cells were observed to recognize both ADAMTSL5 and wheat peptides in psoriasis patients than in healthy controls (**Figures 4D, E**).

### Gluten-free diet (GfD) can improve psoriasis in some patients

**Figure 5:**
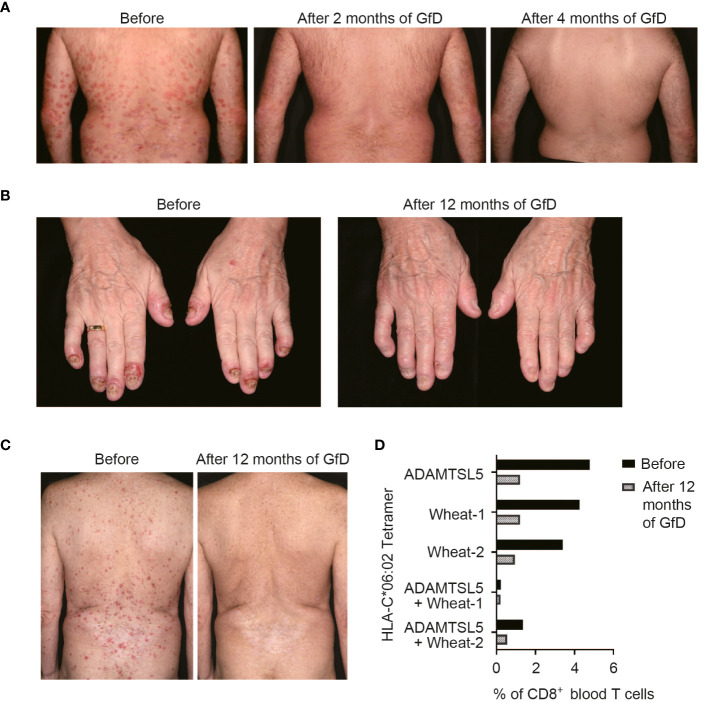
Psoriasis may improve upon a gluten-free diet (GfD). **(A)** Without any change in the therapeutic management, the previously refractory psoriasis cleared within two months upon a GfD in patient #7. **(B)** The formerly treatment-resistant, highly painful DIP-arthritis in patient #10 resolved completely on a GfD, without the need for further treatment. **(C)** Stable psoriasis remission following discontinuation of methotrexate and adalimumab treatment during GfD in patient #12. In this patient, live/CD3^+^CD8^+^CD56^-^ single or double tetramer-stained cells were measured by flow cytometry before and 12 months after starting GfD **(D)**. Patient data are given in **Table 1**.

Our data suggested that wheat antigens may stimulate the psoriatic autoimmune response. As a result, several patients with inadequate response to ongoing systemic psoriasis therapy and lack of therapeutic alternatives opted for a GfD, since gluten-free foods are certified wheat-free. Clinical follow-up showed that seven out of 13 patients experienced a stable improvement in their previously treatment-resistant psoriasis while on a gluten-free diet (**Table 1**, **Figures 5A–C**). Two patients with psoriatic arthritis (#8, 10) that was not sufficiently controlled by systemic treatments became symptom-free (**Figure 5B**). Two patients (#8, 9) experienced a marked psoriasis relapse after dietary errors.

**Table 1:** Patient characteristics and response to gluten-free diet.

Patient #	Sex	Clinical type	Age of disease onset	Gliadin-specific IgG, IgA screen	IgG plus IgA U/ml	IgA U/ml	Age at GFD	Ongoing Treatment	PASI at start of GfD	PASI improvement	PsA improvement	Relapse on dietary error
Non-Responders												
**#1**	M	PsO	23y	pos	11.1	2	43y	Acitretin	15.3	NI	Ø	NR
**#2**	M	PsO	7y	neg	9.8	3.2	30y	FAE	14	NI	Ø	NR
**#3**	F	PsO	32y	pos	ND	ND	64y	anti-TNF	25.4	NI	Ø	NR
**#4**	M	PsO, PsA	51y	pos	12.5	1	55y	anti IL-12/IL-23	17.6	NI	NI	NR
**#5**	M	PsO	17y	pos	10.6	8	47y	Acitretin	12.2	NI	Ø	NR
**#6**	M	PsO	11y	pos	12.5	8.5	36y	Acitretin + FAE	26	NI	Ø	NR
Responders												
**#7**	M	PsO	7y	pos	13.5	12.1	41y	Acitretin	28.5	PASI90	Ø	NR
**#8**	M	PsO, PsA	9y	neg	neg	neg	55y	Acitretin	22	PASI90	Yes	Yes
**#9**	M	PsO, PPPP	28y	pos	17.9	17.9	73y	Aprimelast	15.9	PASI75	Ø	Yes
**#10**	F	PsO, PsA	27y	pos	19.9	17.6	70y	ADA, αIL-12/IL-23, αIL-17 disc.	8.8	PASI75	Yes	NR
**#11**	M	PsO	26y	neg	neg	neg	48y	Acitretin	18.4	PASI90	Ø	NR
**#12**	M	PsO	30y	neg	neg	neg	73	MTX, ADA disc.	15.0	PASI90	Ø	NR
**#13**	M	PsO	16y	neg	neg	neg	39y	Acitretin	14.7	PASI75	Ø	NR

GfD, gluten-free diet; M, male, F, female; y, years; PsO, chronic plaque psoriasis; PsA, psoriatic arthritis; PPPP, palmo-plantar pustular psoriasis; pos, positive; neg, negative; FAE, Fumaric acid esters; MTX, methotrexate; ADA, adalimumab; αIL-12/IL-23, αIL-17, antibodies against IL-12/IL-23 or IL-17; PASI, psoriasis area and severity index; NI, no improvement; PASI75/90, improvement in PASI of ≥75%/≥90%; Ø, not suffering from PsA; ND, not determined; NR, not reported; disc., discontinued. Gliadin IgG and IgA screen before GfD is considered elevated if ≥ 10 U/ml.

The presence of gliadin serum antibodies, which may be elevated in psoriasis patients (35), did not predict the response to GfD (**Table 1**), providing further evidence that CD8^+^ T cells, rather than antibodies, mediate the pathogenic effect of environmental antigens in psoriasis. In patient #12, we had measured the frequency of ADAMTSL5- and wheat-specific CD8^+^ T cells in blood during the GfD by peptide-HLA-C*06:02 tetramer staining. The number of ADAMTSL5- as well as wheat-specific CD8^+^ T cells, and of CD8^+^ T cells that recognize both peptides, declined in the course of dietary wheat avoidance (**Figure 5D**).

## Discussion

Analysis of the cross-reactive potential of the pathogenic psoriatic Vα3S1/Vβ13S1 TCR indicated that a variety of environmental antigens may cross-activate the psoriatic autoimmune response against melanocytes due to TCR polyspecificity. Based on its peptide recognition motif, we identified multiple environmental antigens, all of which ligated the Vα3S1/Vβ13S1 TCR. Several of them were particularly immunogenic for CD8^+^ T cells from psoriasis patients. Remarkably, their origin corresponded to previously suspected psoriasis risk conditions.

The environmental antigens stimulating the Vα3S1/Vβ13S1 TCR and CD8^+^ T cells from psoriasis patients included two peptides from wheat. Detection of dual-specific CD8^+^ blood T cells recognizing both the ADAMTSL5 epitope and wheat peptides directly in patients, by means of peptide-loaded HLA-C*06:02 tetramers, revealed that a cross-reactive T cell response between environmental antigens and self-peptides is a potential pathogenic mechanism in psoriasis. Improvement of psoriasis and psoriatic arthritis observed by us and others (36, 37) in patients under a GfD further supported that wheat peptides may indeed cross-activate the psoriatic autoimmune response. Many wheat peptides resist enzymatic digestion (38), and intact wheat peptides can be detected in blood (39, 40). A T cell-mediated immune response against wheat antigens causes celiac disease and various other pathologic conditions (41), with psoriasis patients being at an increased risk of celiac disease (42). The occasional strikingly high frequency of circulating ADAMTSL5- or wheat peptide-specific CD8^+^ T cells in blood is consistent with other systemic autoimmune diseases such as ANCA vasculitis, which exhibits 6% or more of blood T cells recognizing HLA-tetramer-loaded epitopes of myeloperoxidase, a major ANCA autoantigen (43). In individuals with psoriasis affecting 20% of their body surface area, psoriatic plaques contain approximately 2 x 10^10^ T cells (44). The high frequency of circulating ADAMTSL5-specific T cells detected in psoriasis patients may reflect the fact that the large number of pathogenic T cells infiltrating psoriatic skin lesions may require a continuous T cell supply from the circulation. Indeed, preventing the egress of T cells from secondary lymphoid organs by S1P1 agonists or inhibiting T cell recruitment into the inflammatory sites by integrin (CD11a) blockade effectively ameliorates psoriasis (45, 46). Accordingly, measurement of ADAMTSL5 or wheat peptide-specific blood T cells could be a prognostic biomarker for a potential beneficial effect of a wheat-free diet on disease progression.

The microbiota are thought to play an important role in the development of psoriasis. Firmicutes are significantly overrepresented in skin and gut microbiota of psoriasis patients, and a high intestinal Firmicutes/Bacteroidetes ratio directly correlates with psoriasis severity (47). We identified peptides from *S. agalactiae*, *Clostridium* sp., and *F. plautii* of the bacterial phylum Firmicutes, which activated the ADAMTSL5-specific Vα3S1/Vβ13S1 TCR and stimulated CD8^+^ T cells from psoriasis patients. Another immunogenic peptide was derived from *A. oris*. Periodontitis, associated with a high prevalence of *A. oris* in the oral cavity (48), has been linked to the initiation or propagation of psoriasis and psoriatic arthritis through the immunomodulatory effects of the oral microbiota (49). Continuous exposure to cross-activating microbiota antigens could, therefore, trigger or maintain activation of the psoriatic autoimmune response in psoriasis patients.

Another immunogenic peptide was derived from the baker’s and brewer’s yeast *Saccharomyces cerevisiae*. *S. cerevisiae* is part of the intestinal mycobiome (50) and is present in many foods. As it ferments glucose to alcohol, it is contained in alcoholic beverages, the consumption of which is a potential risk factor for psoriasis (51). Yeast antigens prime long-lasting T_c_17 responses (52). Therefore, *Saccharomyces* antigens released by autolysis of the yeast cells in the gut or during fermentation in alcoholic beverages (53) could induce a cross-reactive autoimmune response against melanocytes, which differentiates into the pathogenic psoriatic T_c_17 phenotype. Remarkably, *S. cerevisiae* antigens drive pathogenic T cell responses in Crohn’s disease (54), which is associated with psoriasis (55) and shares HLA-C*06:02 as a risk gene (56). Avoiding alcohol intake improved psoriasis (36).

Smoking is another independent risk factor of psoriasis (57), and passive tobacco smoke exposure enhances the risk of psoriasis in childhood (58, 59). The cross-activating peptide from the IQ domain-containing protein isoform (IQM3-like isoform) found in tobacco (*Nicotiana tabacum*) and other crops (**Supplementary Table S3**) might trigger the autoimmune response against melanocytes in psoriasis through inhalation of incompletely burnt tobacco proteins, which are transferred to smoke aerosols (60).

*C. trachomatis* infections can induce aberrant autoimmune responses characterized by ankylosing spondylitis, uveitis and inflammatory skin changes resembling psoriasis (61), and psoriatic arthritis patients show an increased prevalence of Chlamydia exposure (62). This attributes a potential pathogenic significance to the chlamydial epitope.

The conditions that either induce or maintain the psoriatic autoimmune response may differ. Infections may break immune tolerance and activate potentially autoreactive naive T cells through strong proinflammatory signals, such as in streptococcal angina, which may precede psoriasis exacerbation (63, 64). Once an autoimmune response has been established, stimulation by environmental peptides mimicking the autoantigen may maintain disease activity under less inflammatory conditions. CD8^+^ memory T cells show a much lower activation threshold than naive CD8^+^ T cells (22, 65, 66), and common proinflammatory gene variants associated with psoriasis could provide the necessary costimulatory innate immune signals maintaining T-cell activation by environmental antigens (3). Avoiding stimulatory environmental antigens, such as through a wheat-free diet, may disrupt the constant generation of cross-reactive pathogenic T cells in lymphoid organs, thereby ameliorating psoriasis.

Overall, we show that due to TCR polyspecificity, multiple environmental antigens from different psoriasis risk conditions converge in the reactivity of an autoreactive psoriatic TCR. The various peptide antigens cross-activating the Vα3S1/Vβ13S1 TCR provide an explanation for the initiation or perpetuation of the T-cell mediated psoriatic autoimmune response against melanocytes by diet, lifestyle, microbiota, and infections. Identification of the environmental antigens through TCR polyspecificity could thus help to develop new approaches to treating psoriasis. Given the multitude of potential triggers, improving psoriasis through lifestyle changes may require a multimodal approach tailored to individual patients. Avoiding wheat products, alcohol, and tobacco might benefit a significant proportion of the huge global psoriasis patient population (2). Thus, gaining insights into the specific response pattern of the autoreactive Vα3S1/Vβ13S1 TCR against environmental antigens may provide a key to a better pathogenetic understanding and practical management of psoriasis. Our data further support that polyspecificity may be a crucial property of TCRs in the induction of human autoimmune diseases.

## Data availability statement

The original contributions presented in the study are included in the article/**Supplementary Material**. Further inquiries can be directed to the corresponding author.

## Ethics statement

The studies involving humans were approved by Ethics Committee of the Medical Faculty, Ludwig-Maximilian-University of Munich. The studies were conducted in accordance with the local legislation and institutional requirements. The participants provided their written informed consent to participate in this study.Written informed consent was obtained from the individual(s) for the publication of any potentially identifiable images or data included in this article.

## Author contributions

TI: Investigation, Methodology, Visualization, Writing – review & editing. YA: Data curation, Formal analysis, Investigation, Methodology, Writing – review & editing. SVu: Data curation, Investigation, Methodology, Validation, Writing – review & editing. JS: Data curation, Investigation, Methodology, Validation, Writing – review & editing. SVo: Investigation, Methodology, Writing – review & editing. AG: Investigation, Methodology, Writing – review & editing. KS: Formal analysis, Investigation, Methodology, Writing – review & editing. GR: Investigation, Methodology, Validation, Writing – review & editing. YP: Methodology, Resources, Writing – review & editing. MD: Methodology, Resources, Writing – review & editing. OH: Investigation, Methodology, Writing – review & editing. MH: Data curation, Investigation, Methodology, Writing – review & editing. BS: Investigation, Methodology, Writing – review & editing. RP: Investigation, Methodology, Writing – review & editing. RA: Investigation, Writing – review & editing. KD: Funding acquisition, Methodology, Supervision, Writing – review & editing. AA: Investigation, Methodology, Project administration, Visualization, Writing – review & editing. JP: Conceptualization, Data curation, Formal analysis, Funding acquisition, Investigation, Methodology, Project administration, Resources, Supervision, Validation, Visualization, Writing – original draft, Writing – review & editing.

## References

[1] Griffiths CEM Armstrong AW Gudjonsson JE Barker J. Psoriasis. Lancet. (2021) 397:1301–15. doi: 10.1016/S0140-6736(20)32549-6 33812489

[2] Parisi R Iskandar IYK Kontopantelis E Augustin M Griffiths CEM Ashcroft DM. National, regional, and worldwide epidemiology of psoriasis: systematic analysis and modelling study. BMJ. (2020) 369:m1590. doi: 10.1136/bmj.m1590 32467098 PMC7254147

[3] Harden JL Krueger JG Bowcock AM. The immunogenetics of Psoriasis: A comprehensive review. J Autoimmun. (2015) 64:66–7310. doi: 10.1016/j.jaut.2015.07.008 26215033 PMC4628849

[4] Nair RP Stuart PE Nistor I Hiremagalore R Chia NV Jenisch S. Sequence and haplotype analysis supports HLA-C as the psoriasis susceptibility 1 gene. Am J Hum Genet. (2006) 78:827–51. doi: 10.1086/503821 PMC1474031 16642438

[5] Chang JC Smith LR Froning KJ Schwabe BJ Laxer JA Caralli LL. CD8+ T cells in psoriatic lesions preferentially use T-cell receptor V beta 3 and/or V beta 13.1 genes. Proc Natl Acad Sci U S A. (1994) 91:9282–6. doi: 10.1073/pnas.91.20.9282 PMC44796 7937756

[6] Menssen A Trommler P Vollmer S Schendel D Albert E Gurtler L. Evidence for an antigen-specific cellular immune response in skin lesions of patients with psoriasis vulgaris. J Immunol. (1995) 155:4078–83. doi: 10.4049/jimmunol.155.8.4078 7561119

[7] Kim SM Bhonsle L Besgen P Nickel J Backes A Held K. Analysis of the paired TCR alpha- and beta-chains of single human T cells. PLoS One. (2012) 7:e37338. doi: 10.1371/journal.pone.0037338 22649519 PMC3359365

[8] Di Meglio P Villanova F Navarini AA Mylonas A Tosi I Nestle FO. Targeting CD8(+) T cells prevents psoriasis development. J Allergy Clin Immunol. (2016) 138:274–6.e6. doi: 10.1016/j.jaci.2015.10.046 26782974

[9] Prinz JC Vollmer S Boehncke WH Menssen A Laisney I Trommler P. Selection of conserved TCR VDJ rearrangements in chronic psoriatic plaques indicates a common antigen in psoriasis vulgaris. Eur J Immunol. (1999) 29:3360–8. doi: 10.1002/(SICI)1521-4141(199910)29:10<3360::AID-IMMU3360>3.0.CO;2-G 10540348

[10] Henseler T Christophers E. Psoriasis of early and late onset: characterization of two types of psoriasis vulgaris. J Am Acad Dermatol. (1985) 13:450–6. doi: 10.1016/S0190-9622(85)70188-0 4056119

[11] Lonnberg AS Skov L Skytthe A Kyvik KO Pedersen OB Thomsen SF. Heritability of psoriasis in a large twin sample. Brit J Dermatol. (2013) 169:412–6. doi: 10.1111/bjd.12375 23574549

[12] Grjibovski AM Olsen AO Magnus P Harris JR. Psoriasis in Norwegian twins: contribution of genetic and environmental effects. J Eur Acad Dermatol Venereol. (2007) 21:1337–43. doi: 10.1111/j.1468-3083.2007.02268.x 17958839

[13] Arakawa A Siewert K Stohr J Besgen P Kim SM Ruhl G. Melanocyte antigen triggers autoimmunity in human psoriasis. J Exp Med. (2015) 212:2203–12. doi: 10.1084/jem.20151093 PMC4689169 26621454

[14] Arakawa A Reeves E Vollmer S Arakawa Y He M Galinski A. ERAP1 controls the autoimmune response against melanocytes in psoriasis by generating the melanocyte autoantigen and regulating its amount for HLA-C*06:02 presentation. J Immunol. (2021) 207:2235–44. doi: 10.4049/jimmunol.2100686 PMC7611875 34580106

[15] Cheuk S Schlums H Gallais Serezal I Martini E Chiang SC Marquardt N. CD49a expression defines tissue-resident CD8(+) T cells poised for cytotoxic function in human skin. Immunity. (2017) 46:287–300. doi: 10.1016/j.immuni.2017.01.009 28214226 PMC5337619

[16] Hijnen D Knol EF Gent YY Giovannone B Beijn SJ Kupper TS. CD8(+) T cells in the lesional skin of atopic dermatitis and psoriasis patients are an important source of IFN-gamma, IL-13, IL-17, and IL-22. J Invest Dermatol. (2013) 133:973–9. doi: 10.1016/j.immuni.2017.01.009 PMC3835628 23223131

[17] Prinz JC. The Woronoff ring in psoriasis and the mechanisms of postinflammatory hypopigmentation. Acta Derm Venereol. (2020) 100:adv00031. doi: 10.2340/00015555-3385 31971604 PMC9128907

[18] Ma F Plazyo O Billi AC Tsoi LC Xing X Wasikowski R. Single cell and spatial sequencing define processes by which keratinocytes and fibroblasts amplify inflammatory responses in psoriasis. Nat Comm. (2023) 14:3455. doi: 10.1038/s41467-023-39020-4 PMC10261041 37308489

[19] Ortega C Fernandez AS Carrillo JM Romero P Molina IJ Moreno JC. IL-17-producing CD8+ T lymphocytes from psoriasis skin plaques are cytotoxic effector cells that secrete Th17-related cytokines. J Leukoc Biol. (2009) 86:435–43. doi: 10.1189/JLB.0109046 19487306

[20] Birnbaum ME Mendoza JL Sethi DK Dong S Glanville J Dobbins J. Deconstructing the peptide-MHC specificity of T cell recognition. Cell. (2014) 157:1073–87. doi: 10.1016/j.cell.2014.03.047 PMC4071348 24855945

[21] Nelson RW Beisang D Tubo NJ Dileepan T Wiesner DL Nielsen K. T cell receptor cross-reactivity between similar foreign and self peptides influences naive cell population size and autoimmunity. Immunity. (2015) 42:95–107. doi: 10.1016/j.immuni.2014.12.022 25601203 PMC4355167

[22] Sewell AK. Why must T cells be cross-reactive? Nat Rev Immunol. (2012) 12:669–77. doi: 10.1038/nri3279 PMC7097784 22918468

[23] Yang Q Liu H Qu L Fu X Yu Y Yu G. Investigation of 20 non-HLA (human leucocyte antigen) psoriasis susceptibility loci in Chinese patients with psoriatic arthritis and psoriasis vulgaris. Brit J Dermatol. (2013) 168:1060–5. doi: 10.1111/bjd.12142 23252691

[24] Siewert K Malotka J Kawakami N Wekerle H Hohlfeld R Dornmair K. Unbiased identification of target antigens of CD8+ T cells with combinatorial libraries coding for short peptides. Nat Med. (2012) 18:824–8. doi: 10.1038/nm.2720 22484809

[25] Falk K Rotzschke O Stevanovic S Jung G Rammensee HG. Allele-specific motifs revealed by sequencing of self-peptides eluted from MHC molecules. Nature. (1991) 351:290–6. doi: 10.1038/351290a0 1709722

[26] Tazi Ahnini R Camp NJ Cork MJ Mee JB Keohane SG Duff GW. Novel genetic association between the corneodesmosin (MHC S) gene and susceptibility to psoriasis. H Mol Gen. (1999) 8:1135–40. doi: 10.1093/hmg/8.6.1135 10332047

[27] Wolfl M Kuball J Ho WY Nguyen H Manley TJ Bleakley M. Activation-induced expression of CD137 permits detection, isolation, and expansion of the full repertoire of CD8+ T cells responding to antigen without requiring knowledge of epitope specificities. Blood. (2007) 110:201–10. doi: 10.1182/blood-2006-11-056168 PMC1896114 17371945

[28] Altman JD Moss PA Goulder PJ Barouch DH McHeyzer-Williams MG Bell JI. Phenotypic analysis of antigen-specific T lymphocytes. Science. (1996) 274:94–6. doi: 10.1126/science.274.5284.94 21690331

[29] Bodinier M Peyrat MA Tournay C Davodeau F Romagne F Bonneville M. Efficient detection and immunomagnetic sorting of specific T cells using multimers of MHC class I and peptide with reduced CD8 binding. Nat Med. (2000) 6:707–10. doi: 10.1038/76292 10835691

[30] Jansen D Ramnoruth N Loh KL Rossjohn J Reid HH Nel HJ. Flow cytometric clinical immunomonitoring using peptide-MHC class II tetramers: optimization of methods and protocol development. Front Immunol. (2018) 9:8. doi: 10.3389/fimmu.2018.00008 29403492 PMC5786526

[31] Cunningham BC Wells JA. High-resolution epitope mapping of hGH-receptor interactions by alanine-scanning mutagenesis. Science. (1989) 244:1081–5. doi: 10.1126/science.2471267 2471267

[32] Rasmussen M Harndahl M Stryhn A Boucherma R Nielsen LL Lemonnier FA. Uncovering the peptide-binding specificities of HLA-C: a general strategy to determine the specificity of any MHC class I molecule. J Immunol. (2014) 193:4790–802. doi: 10.4049/jimmunol.1401689 PMC4226424 25311805

[33] Mobbs JI Illing PT Dudek NL Brooks AG Baker DG Purcell AW. The molecular basis for peptide repertoire selection in the human leucocyte antigen (HLA) C*06:02 molecule. J Biol Chem. (2017) 292:17203–15. doi: 10.1074/jbc.M117.806976 PMC5655500 28855257

[34] Di Marco M Schuster H Backert L Ghosh M Rammensee HG Stevanovic S. Unveiling the peptide motifs of HLA-C and HLA-G from naturally presented peptides and generation of binding prediction matrices. J Immunol. (2017) 199:2639–51. doi: 10.1074/jbc.M117.806976 28904123

[35] Kolchak NA Tetarnikova MK Theodoropoulou MS Michalopoulou AP Theodoropoulos DS. Prevalence of antigliadin IgA antibodies in psoriasis vulgaris and response of seropositive patients to a gluten-free diet. J Multidiscip Healthc. (2018) 11:13–9. doi: 10.2147/JMDH.S122256 PMC5747961 29343966

[36] Afifi L Danesh MJ Lee KM Beroukhim K Farahnik B Ahn RS. Dietary behaviors in psoriasis: patient-reported outcomes from a U.S. National survey. Dermatol Ther (Heidelb). (2017) 7:227–42. doi: 10.1007/s13555-017-0183-4 PMC5453925 28526915

[37] Michaelsson G Gerden B Hagforsen E Nilsson B Pihl-Lundin I Kraaz W. Psoriasis patients with antibodies to gliadin can be improved by a gluten-free diet. Brit J Dermatol. (2000) 142:44–51. doi: 10.1046/j.1365-2133.2000.03240.x 10651693

[38] Marti T Molberg O Li Q Gray GM Khosla C Sollid LM. Prolyl endopeptidase-mediated destruction of T cell epitopes in whole gluten: chemical and immunological characterization. J Pharmacol Exp Ther. (2005) 312:19–26. doi: 10.1124/jpet.104.073312 15358813

[39] Macdonald TT Monteleone G. Immunity, inflammation, and allergy in the gut. Science. (2005) 307:1920–5. doi: 10.1126/science.1106442 15790845

[40] Drago S El Asmar R Di Pierro M Grazia Clemente M Tripathi A Sapone A. Gliadin, zonulin and gut permeability: Effects on celiac and non-celiac intestinal mucosa and intestinal cell lines. Scand J Gastroenterol. (2006) 41:408–19. doi: 10.1080/00365520500235334 16635908

[41] Manzel A Muller DN Hafler DA Erdman SE Linker RA Kleinewietfeld M. Role of "Western diet" in inflammatory autoimmune diseases. Curr Allergy Asthma Rep. (2014) 14:404. doi: 10.1007/s11882-013-0404-6 24338487 PMC4034518

[42] Bhatia BK Millsop JW Debbaneh M Koo J Linos E Liao W. Diet and psoriasis, part II: celiac disease and role of a gluten-free diet. J Am Acad Dermatol. (2014) 71:350–8. doi: 10.1016/j.jaad.2014.03.017 PMC4104239 24780176

[43] Free ME Stember KG Hess JJ McInnis EA Lardinois O Hogan SL. Restricted myeloperoxidase epitopes drive the adaptive immune response in MPO-ANCA vasculitis. J Autimmun. (2020) 106:102306. doi: 10.1016/j.jaad.2014.03.017 PMC6930338 31383567

[44] Krueger JG Bowcock A. Psoriasis pathophysiology: current concepts of pathogenesis. Ann Rheum Dis. (2005) 64 Suppl 2:ii30–6. doi: 10.1136/ard.2004.031120 PMC1766865 15708932

[45] Vugmeyster Y Kikuchi T Lowes MA Chamian F Kagen M Gilleaudeau P. Efalizumab (anti-CD11a)-induced increase in peripheral blood leukocytes in psoriasis patients is preferentially mediated by altered trafficking of memory CD8+ T cells into lesional skin. Clin Immunol. (2004) 113:38–46. doi: 10.1136/ard.2004.031120 15380528

[46] Vaclavkova A Chimenti S Arenberger P Hollo P Sator PG Burcklen M. Oral ponesimod in patients with chronic plaque psoriasis: a randomised, double-blind, placebo-controlled phase 2 trial. Lancet. (2014) 384:2036–45. doi: 10.1016/S0140-6736(14)60803-5 25127208

[47] Todberg T Kaiser H Zachariae C Egeberg A Halling AS Skov L. Characterization of the oral and gut microbiota in patients with psoriatic diseases: A systematic review. Acta Derm Venereol. (2021) 101:adv00512. doi: 10.2340/00015555-3882 34263334 PMC9413811

[48] Vielkind P Jentsch H Eschrich K Rodloff AC Stingu CS. Prevalence of Actinomyces spp. in patients with chronic periodontitis. Int J Med Microbiol. (2015) 305:682–8. doi: 10.1016/j.ijmm.2015.08.018 26324012

[49] Egeberg A Mallbris L Gislason G Hansen PR Mrowietz U. Risk of periodontitis in patients with psoriasis and psoriatic arthritis. J Eur Acad Dermatol Venereol. (2017) 31:288–93. doi: 10.1111/jdv.13814 27439545

[50] Hallen-Adams HE Suhr MJ. Fungi in the healthy human gastrointestinal tract. Virulence. (2017) 8:352–8. doi: 10.1080/21505594.2016.1247140 PMC5411236 27736307

[51] Svanstrom C Lonne-Rahm SB Nordlind K. Psoriasis and alcohol. Psoriasis (Auckl). (2019) 9:75–9. doi: 10.2147/PTT.S164104 PMC6709030 31687362

[52] Nanjappa SG McDermott AJ Fites JS Galles K Wuthrich M Deepe GS Jr.. Antifungal Tc17 cells are durable and stable, persisting as long-lasting vaccine memory without plasticity towards IFNgamma cells. PLoS Pathog. (2017) 13:e1006356. doi: 10.1371/journal.ppat.1006356 28542595 PMC5456400

[53] Alexandre H Heintz D Chassagne D Guilloux-Benatier M Charpentier C Feuillat M. Protease A activity and nitrogen fractions released during alcoholic fermentation and autolysis in enological conditions. J Ind Microbiol Biotechnol. (2001) 26:235–40. doi: 10.1038/sj.jim.7000119 11464273

[54] Martini GR Tikhonova E Rosati E DeCelie MB Sievers LK Tran F. Selection of cross-reactive T cells by commensal and food-derived yeasts drives cytotoxic T(H)1 cell responses in Crohn's disease. Nat Med. (2023) 29:2602–14. doi: 10.1038/s41591-023-02556-5 PMC10579100 37749331

[55] Bezzio C Della Corte C Vernero M Di Luna I Manes G Saibeni S. Inflammatory bowel disease and immune-mediated inflammatory diseases: looking at the less frequent associations. Therap Adv Gastroenterol. (2022) 15:17562848221115312. doi: 10.1177/17562848221115312 PMC9340394 35924080

[56] Goyette P Boucher G Mallon D Ellinghaus E Jostins L Huang H. High-density mapping of the MHC identifies a shared role for HLA-DRB1*01:03 in inflammatory bowel diseases and heterozygous advantage in ulcerative colitis. Nat Genet. (2015) 47:172–9. doi: 10.1038/ng.3176 PMC4310771 25559196

[57] Armstrong AW Harskamp CT Dhillon JS Armstrong EJ. Psoriasis and smoking: a systematic review and meta-analysis. Brit J Dermatol. (2014) 170:304–14. doi: 10.1111/bjd.12670 24117435

[58] Groot J Nybo Andersen AM Blegvad C Pinot de Moira A Skov L. Prenatal, infantile, and childhood tobacco exposure and risk of pediatric psoriasis in the Danish National Birth Cohort offspring. J Am Acad Dermatol. (2020) 83:1625–32. doi: 10.1016/j.jaad.2019.09.038 31973955

[59] Mahid SS Minor KS Stromberg AJ Galandiuk S. Active and passive smoking in childhood is related to the development of inflammatory bowel disease. Inflammation Bowel Dis. (2007) 13:431–8. doi: 10.1002/ibd.20070 17206676

[60] Koethe SM Nelson KE Becker CG. Activation of the classical pathway of complement by tobacco glycoprotein (TGP). J Immunol. (1995) 155:826–35. doi: 10.4049/jimmunol.155.2.826 7608560

[61] Stavropoulos PG Soura E Kanelleas A Katsambas A Antoniou C. Reactive arthritis. J Eur Acad Dermatol Venereol. (2015) 29:415–24. doi: 10.1111/jdv.12741 25199646

[62] Thrastardottir T Love TJ. Infections and the risk of psoriatic arthritis among psoriasis patients: a systematic review. Rheumatol Int. (2018) 38:1385–97. doi: 10.1007/s00296-017-3873-4 29124396

[63] Mills KH. TLR-dependent T cell activation in autoimmunity. Nat Rev Immunol. (2011) 11:807–22. doi: 10.1038/nri3095 22094985

[64] Gudjonsson JE Thorarinsson AM Sigurgeirsson B Kristinsson KG Valdimarsson H. Streptococcal throat infections and exacerbation of chronic plaque psoriasis: a prospective study. Brit J Dermatol. (2003) 149:530–4. doi: 10.1046/j.1365-2133.2003.05552.x 14510985

[65] Curtsinger JM Lins DC Mescher MF. CD8+ memory T cells (CD44high, Ly-6C+) are more sensitive than naive cells to (CD44low, Ly-6C-) to TCR/CD8 signaling in response to antigen. J Immunol. (1998) 160:3236–43. doi: 10.4049/jimmunol.160.7.3236 9531279

[66] Veiga-Fernandes H Walter U Bourgeois C McLean A Rocha B. Response of naive and memory CD8+ T cells to antigen stimulation in *vivo* . Nat Immunol. (2000) 1:47–53. doi: 10.1038/76907 10881174

