# Supplementary material for: Multiple environmental antigens may trigger autoimmunity in psoriasis through T-cell receptor polyspecificity
Source: Front Immunol. 2024 Mar 8;15:1374581. doi: 10.3389/fimmu.2024.1374581 (PMC10958380; doi:10.3389/fimmu.2024.1374581)
Supplement: Supplementary file 1 [file DataSheet_1.pdf]

“Multiple environmental antigens may trigger autoimmunity in psoriasis through T-cell receptor polyspecificity”

T. Ishimoto et al.

Supplementary material:

- **Supplementary Figures S1-S4**
- **Supplementary Tables S1-S3**

Figure S1

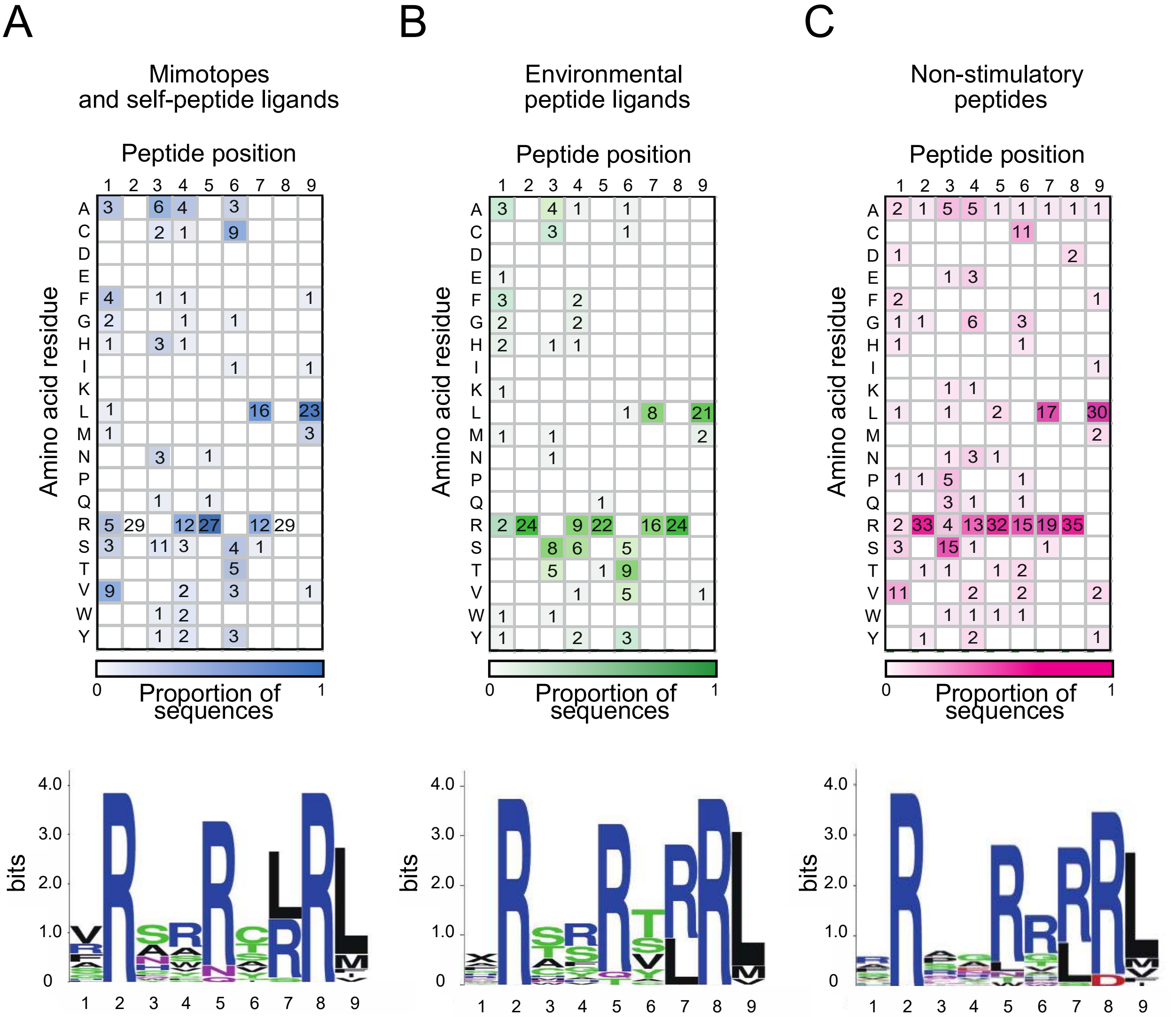

Figure S1. Amino acid preferences define the Vα3S1/Vβ13S1 TCR recognition motif. Amino acid preferences by position shown as heatmaps or sequence logos created by the WebLogo tool at https://weblogo.berkeley.edu/logo.cgi for (**A**) mimotopes and previously identified peptide ligands (table S1), (**B**) naturally occurring stimulatory environmental peptide ligands (table S2), and (**C**) non-stimulatory environmental peptides selected according to the Vα3S1/Vβ13S1 TCR recognition motif (table S2). Data summarize nonamers and octamers lacking the NH_2-terminal amino acid at P1 of nonamers.

Figure S2

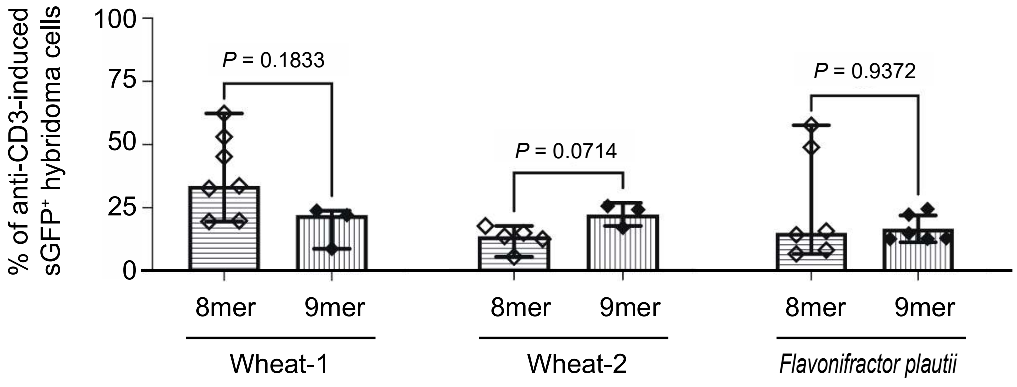

Figure S2. Analysis of Vα3S1/Vβ13S1-TCR hybridoma stimulation by antigenic octamers and nonamers. Response to stimulation of the Vα3S1/Vβ13S1-TCR hybridoma by co-culture with COS-7 cells cotransfected with HLA-C*06:02 and plasmids encoding either wheat-1, wheat-2, or *Flavonifractor plautii* octamers or nonamers. Induction of sGFP was determined by flow cytometry after 24h. Data summarize duplicates or triplicates. Results are given as percentage of CD3 antibody-stimulated GFP^+ hybridoma cells, which indicates the approximate overall activatable TCR hybridoma cells. Bars represent median and interquartile values. Two-group comparison was performed using a non-parametric two-sided Mann-Whitney U test for unpaired continuous variables.

Figure S3

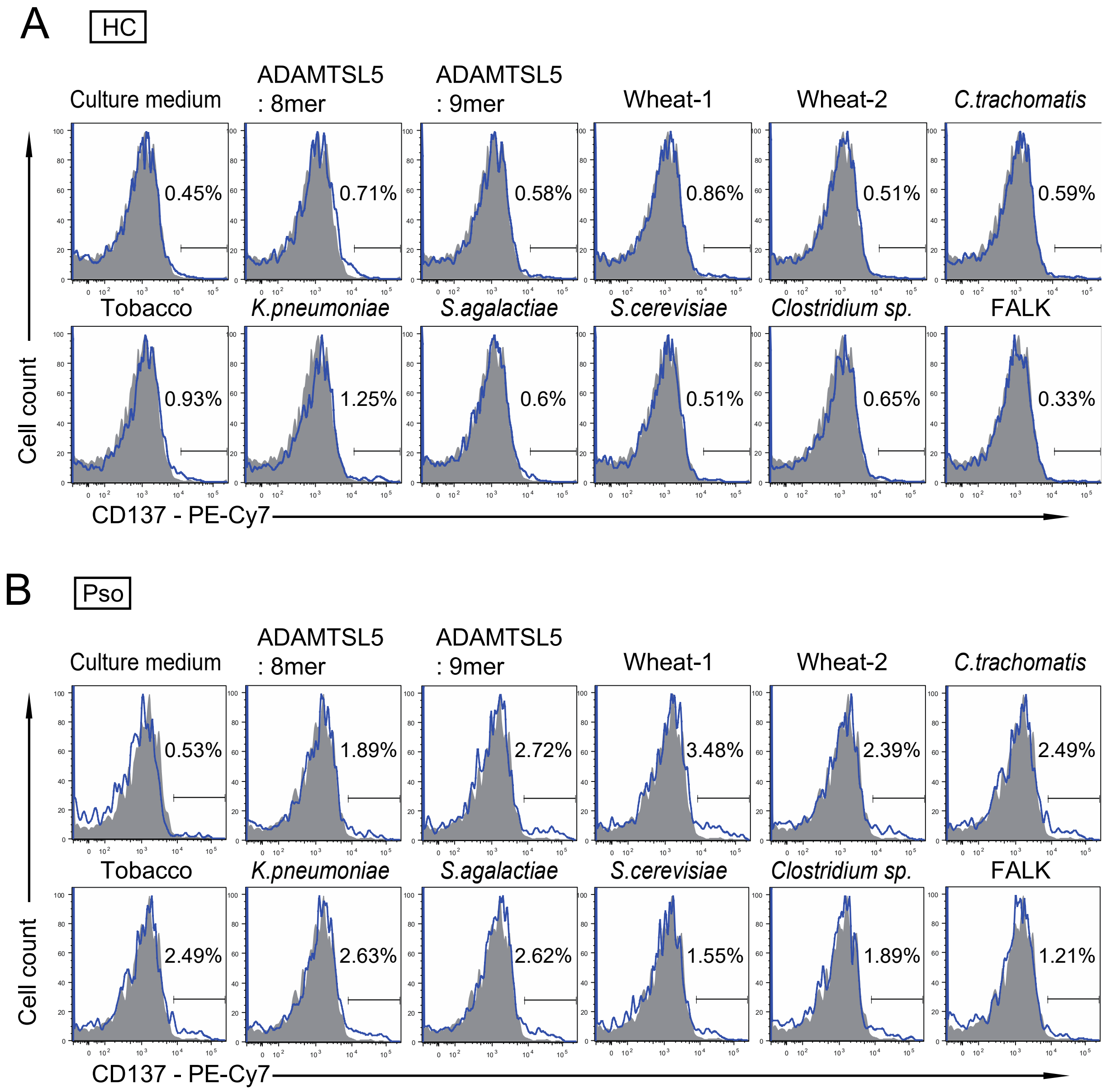

Figure S3. Representative flow cytometry analysis of stimulation-induced CD137 expression on CD8⁺ T cells. (**A**) Healthy control (HC); (**B**) Psoriasis patient (Pso). PBMC were stimulated with synthetic peptides for 24h. CD137 expression was determined on lymphocytes gated for the expression of CD8. Isotype control is given as grey background.

Figure S4

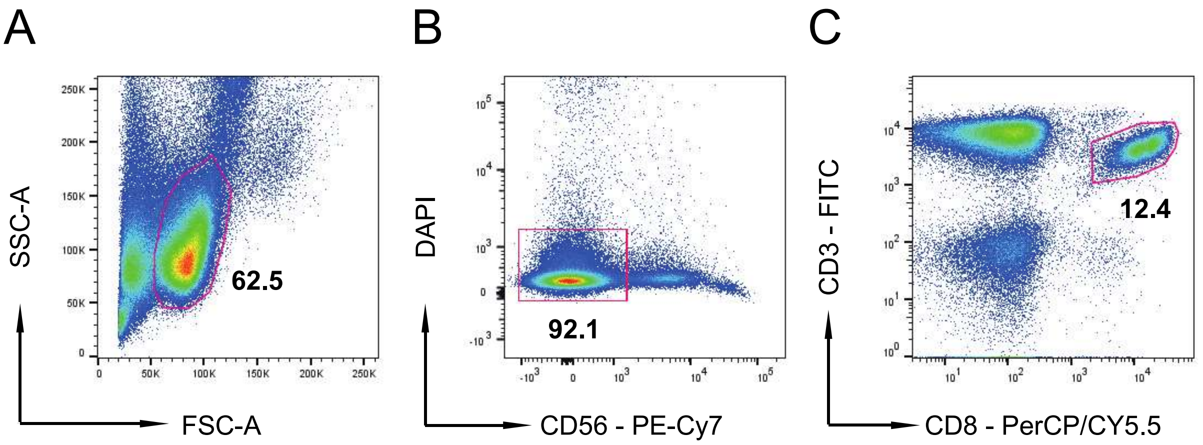

Figure S4. Gating strategy for the identification of Live/CD3^{+}/CD8^{+}/CD56^{-} peptide-HLA-C*06:02 tetramer^{+} cells. PBMC from HLA-C*06:02^{+} donors were stained with 4',6-diamidino-2-phenylindole (DAPI) and monoclonal antibodies against the cell surface markers CD3 (FITC), CD8 (PerCP/CY5.5), CD56 (PE-Cy7), and BV421- or PE-labelled peptide-HLA-C*06:02 tetramers. Lymphocytes were determined by forward (FSC) and side scatter (SSC) gating (**A**). A second gate (**B**) discriminated live from dead cells and excluded CD56^{+} cells to minimize the impact of Killer cell Ig-like receptors (KIR) of natural killer cells on tetramer binding. (**C**) Subsequently, live/CD56^{-} cells were gated on the CD3^{+}CD8^{+} double-positive cells and analyzed for peptide-HLA-C*06:02 tetramer staining (**Figure 4B**).

Table S1 **Sequence of peptide antigens used to determine the recognition motif of the Vα3S1/Vβ13S1 TCR given in Fig. S1**

Stimulatory mimotopes and human peptides	Amino acid position 1	2	3	4	5	6	7	8	9	
Mimotope 1	M	R	S	H	R	Y	L	R	L	
Mimotope 2	G	R	N	A	R	V	R	R	L	
Mimotope 3	G	R	A	S	R	T	R	R	L	
Mimotope 4	R	R	N	W	N	Y	R	R	L	
Mimotope 5	F	R	W	Y	R	V	R	R	L	
Mimotope 6	F	R	C	R	Q	Y	R	R	L	
Mimotope 7	F	R	H	R	R	T	L	R	L	
Mimotope 8	F	R	S	Y	R	T	R	R	L	
Mimotope 9	R	R	Y	F	R	C	L	R	M	
Mimotope 10	R	R	N	A	R	V	R	R	L	
Mimotope 11	V	R	C	W	R	G	R	R	L	
ADAMTSL5 57-65	V	R	S	R	R	C	L	R	L	
THEM6 105-113	A	R	H	R	R	S	L	R	L	
RASSF10 44-52	R	R	Q	R	R	S	R	R	L	
HEPACAM 9-17	S	R	A	S	R	A	L	R	L	
ASH1L 2422-2430	A	R	S	V	R	T	R	R	L	
C2CD4B 193-201	L	R	A	G	R	S	R	R	L	
C16orf1	S	R	A	V	R	A	L	R	L	
Uncharacterized protein	S	R	A	A	R	S	R	R	L	
YB-1 193-201	H	R	H	S	R	I	L	R	L	
IL-24del3 193-201	R	R	F	C	R	T	S	R	M	
ADAMTSL5-A1 57-65	A	R	S	R	R	C	L	R	L	Alanin exchange
ADAMTSL5-A3 57-65	V	R	A	R	R	C	L	R	L	Alanin exchange
ADAMTSL5-A4 57-65	V	R	S	A	R	C	L	R	L	Alanin exchange
ADAMTSL5-A6 57-65	V	R	S	R	R	A	L	R	L	Alanin exchange
ADAMTSL5-V9 57-65	V	R	S	R	R	C	L	R	V	Alternative anchor residue
ADAMTSL5-F9 57-65	V	R	S	R	R	C	L	R	F	Alternative anchor residue
ADAMTSL5-I9 57-65	V	R	S	R	R	C	L	R	I	Alternative anchor residue
ADAMTSL5-M9 57-65	V	R	S	R	R	C	L	R	M	Alternative anchor residue

Non-stimulatory peptides	Amino acid position 1	2	3	4	5	6	7	8	9	
AG3 Corynebact durum	S	R	T	R	R	T	R	R	M	
AG13 Beta vulgaris	P	R	N	A	R	V	R	R	L	
AG17 Triticum aest.	R	R	P	A	R	R	R	R	L	
AG21 Lactobac. Bifidum	F	R	S	Y	W	P	R	R	L	
AG23 Turicella otidis	L	R	W	Y	T	V	R	R	L	
AG26 Triticum aest.	H	R	R	G	R	Q	R	R	L	
AG27 Triticum aest.	V	R	A	G	R	G	L	R	L	
AG28 Triticum aest	G	R	R	E	R	R	R	R	L	
AG29 Triticum aest	D	R	P	R	R	R	R	R	L	
AG30 Triticum aest	F	R	S	N	R	R	L	R	L	
AG31 Triticum aest	A	R	S	A	R	R	R	R	L	
AG32 Triticum aest	A	R	A	A	R	R	R	R	L	
AG33 Triticum aest	R	R	R	R	R	R	L	R	L	
AG34 Triticum aest		R	P	E	R	R	R	R	L	
AG35 Triticum aest		R	P	G	R	R	R	R	L	
AG36 Triticum aest		R	P	V	R	R	R	R	L	
AG37 Triticum aest		R	A	G	R	G	L	R	L	
AG38 Triticum aest		R	S	N	R	R	L	R	L	
AG39 Triticum aest		R	A	A	R	R	R	R	L	
AG41 Triticum aest		R	S	N	R	R	L	R	L	
AG43 Strep. pyogenes		R	Q	K	R	H	R	R	V	
AG44 Mycobact. Bovis		R	Q	W	R	A	L	R	M	
AG49 Candida albicans		R	A	S	R	G	S	R	L	
AG50 Candida albicans		R	R	V	R	W	L	R	V	
AG51 Candida albicans		R	E	Q	R	C	R	R	F	
AG52 Candida albicans		R	L	E	N	T	R	R	I	
AG 55 Strept. pyogenes	S	R	Q	G	L	R	R	D	L	
AG54 Strep. pyogenes	S	R	K	G	L	R	R	D	L	
ADAMTSL5 ALA2	V	A	S	R	R	C	L	R	L	Alanin exchange
ADAMTSL5 ALA5	V	R	S	R	A	C	L	R	L	Alanin exchange
ADAMTSL5 ALA7	V	R	S	R	R	C	A	R	L	Alanin exchange
ADAMTSL5 ALA8	V	R	S	R	R	C	L	A	L	Alanin exchange
ADAMTSL5 ALA9	V	R	S	R	R	C	L	R	A	Alanin exchange
ADAMTSL5 P2G	V	G	S	R	R	C	L	R	L	Alternative anchor residue
ADAMTSL5 P2T	V	T	S	R	R	C	L	R	L	Alternative anchor residue
ADAMTSL5 P2P	V	P	S	R	R	C	L	R	L	Alternative anchor residue
ADAMTSL5 P2Y	V	Y	S	R	R	C	L	R	L	Alternative anchor residue
ADAMTSL5 YP9	V	R	S	R	R	C	L	R	Y	Alternative anchor residue

Red letters designate amino acid exchange in the original peptide sequence

Table S2: Environmental Peptide Antigens selected for Vα3S1/Vβ13S1 TCR stimulation

	Organism	Protein and Link	Test result	Peptide sequence 1	2	3	4	5	6	7	8	9	Primers for cloning 5'-3'
**STIMULATORY PEPTDIES**	*Serpula lacrymans var. lacrymans*<br>Fungi	Hypothetical protein SERLADRAFT_432168<br>Link	Tested<br>Positive	H	R	T	R	R	T	R	R	L	Forward cacc atg cac cgt acc agg cgt acc cgc agg ctt tga<br>Reverse tca aag cct gcg ggt acg cct ggt acg gtg
	Mycobacterium tuberculosis<br>Bacteria	Phenolpthiocerol synthesis type-I polyketide synthase PPSA<br>Link	Tested<br>Positive	R	R	T	R	R	T	R	R	L	Forward cacc atg cgc cga acc cgc cga acc cgc cgg ctc tga<br>Reverse tca gag ccg gcg ggt tcg gcg ggt tcg gcg
	Chlamydia trachomatis<br>Bacteria	Uncharacterised protein<br>Link	Tested<br>Positive	F	R	S	Y	R	V	R	R	L	Forward cacc atg ttc cga tcc tat cgt gtt aga aga ttg tga<br>Reverse tca caa tct tct aac acg ata gga tcg gaa
	Nicotiana tabacum<br>Crop	IQM3-like isoform<br>Link	Tested<br>Positive	Y	R	S	Y	R	T	R	R	M	Forward cacc atg tac agg agc tat cgt act cga cgc atg tga<br>Reverse tca cat gcg tcg agt acg ata gct cct gta
	Aspergillus niger<br>Fungi	Unnamed protein product<br>Link	Tested<br>Positive	G	R	A	S	R	S	R	R	L	Forward cacc atg gga agg gct tca aga tca cga aga ctg tga<br>Reverse tca cag tct tcg tga tct tga agc cct tcc
	Flavonifractor plautii<br>Microbiota	Hypothetical protein<br>Link	Tested<br>Positive	E	R	A	A	R	T	R	R	L	Forward cacc atg gaa cgt gct gca aga aca cgg aga ctt tga<br>Reverse tca aag tct ccg tgt tct tgc agc acg ttc
	Flavonifractor plautii<br>Microbiota	Hypothetical protein, octamer<br>Link	Tested<br>Positive		R	A	A	R	T	R	R	L	Forward cacc atg cgt gct gca aga aca cgg aga ctt tga<br>Reverse tca aag tct ccg tgt tct tgc agc acg
	Klebsiella pneumoniae<br>Bacteria	Tail protein<br>Link	Tested<br>Positive	K	R	S	R	R	V	L	R	L	Forward cacc atg aaa cgc agt cgt cgt gtg ctg cgt tta tga<br>Reverse tca taa acg cag cac acg acg act gcg ttt
	Malus domestica<br>Crop	Uncharacterized protein LOC103417966<br>Link	Tested<br>Positive	A	R	S	F	R	S	L	R	L	Forward cacc atg gca cgt tcg ttc cgt tcg ctc cgt tta tga<br>Reverse tca taa acg gag cga acg gaa cga acg tgc
	Deltaproteobacteria Bacterium<br>Bacteria	LysR family transcriptional regulator<br>Link	Tested<br>Positive	A	R	S	S	R	S	L	R	L	Forward cacc atg gca cgt tcg tcc cgt tcg ctc cgt tta tga<br>Reverse tca taa acg gag cga acg gga cga acg tgc
	Nocardia brasiliensis<br>Bacteria	Hypothetical protein NBRGN_050_00490<br>Link	Tested<br>Positive	R	R	T	R	R	S	R	R	L	Forward cacc atg cgc cgc acg cgg cgg tcc agg cgc ctc tga<br>Reverse tca gag gcg cct gga ccg ccg cgt gcg gcg
	Pseudomonas syringae<br>Bacteria	Hypothetical protein<br>Link	Tested<br>Positive	M	R	W	F	R	V	R	R	L	Forward cacc atg atg agg tgg ttt cga gtc cgc cga ttg tga<br>Reverse tca caa tcg gcg gac tcg aaa cca cct cat
	Spinacia oleracea<br>Crop	Hypothetical protein SOVF_054900 isoform A<br>Link	Tested<br>Positive	H	R	N	H	R	Y	L	R	L	Forward cacc atg cat cgg aac cac cgt tac ctc cgc tta tga<br>Reverse tca taa gcg gag gta acg gtg gtt ccg atg
	Pectobacterium atrosepticum<br>Bacteria	CDP-diglyceride synthetase<br>Link	Tested<br>Positive	W	R	H	S	R	A	L	R	L	Forward cacc atg tgg cgt cat tca cgc gca ttg cgg ctc tga<br>Reverse tca gag ccg caa tgc gcg tga atg acg cca
	Burkholderia lata<br>Bacteria	Hypothetical protein<br>Link	Tested<br>Positive	G	R	T	S	R	T	R	R	L	Forward cacc atg ggc cgg aca tcg cgc aca cgg cga ctc tga<br>Reverse tca gag tcg ccg tgt gcg cga tgt ccg gcc
	Desulfovibrio aminophilus<br>Bacteria	Hypothetical protein<br>Link	Tested<br>Positive	L	R	A	S	R	T	R	R	L	Forward cacc atg cgc gcc agc cgc aca cgc cgc ctg tga<br>Reverse tca cag gcg gcg tgt gcg gct ggc gcg
	Triticum aestivum wheat 1<br>Crop	RGC1B<br>Link	Tested<br>Positive	L	R	M	R	R	C	R	R	M	Forward cacc atg ctc agg atg aga aga tgt cgg cgt atg tga<br>Reverse tca cat acg ccg aca tct tct cat cct gag
	Triticum aestivum, wheat 1<br>Crop	RGC1B, octamer<br>Link	Tested<br>Positive		R	M	R	R	C	R	R	M	Forward cacc atg agg atg aga aga tgt cgg cgt atg tga<br>Reverse tca cat acg ccg aca tct tct cat cct
	Triticum aestivum wheat 2<br>Crop	Hypothetical protein CFC21_050201<br>Link	Tested<br>Positive	V	R	A	G	R	V	L	R	V	Forward cacc atg gtc cgc gcc ggc cgc gtc ctc cgc gtc tga<br>Reverse tca gac gcg gag gac gcg gcc ggc gcg gac
	Triticum aestivum, wheat 2<br>Crop	Hypothetical protein CFC21_050201, octamer<br>Link	Tested<br>Positive		R	A	G	R	V	L	R	V	Forward cacc atg cgc gcc ggc cgc gtc ctc cgc gtc tga<br>Reverse tca gac gcg gag gac gcg gcc ggc gcg
	Clostridium sp.<br>Microbiota	Alpha-galactosidase<br>Link	Tested<br>Positive		R	S	V	R	T	R	R	L	Forward cacc atg cgg tcc gtc aga acg aga agg ctg tga<br>Reverse tca cag cct tct cgt tct gac gga ccg
	Streptococcus agalactiae<br>Microbiota	ABC transporter ATP-binding protein<br>Link	Tested<br>Positive		R	C	S	R	V	L	R	L	Forward cacc atg aga tgt tcc cga gta cta cga tta tga<br>Reverse tca taa tcg tag tac tcg gga aca tct
	Actinomyces oris<br>Microbiota	Putative serine/threonine-protein kinase HSL1<br>Link	Tested<br>Positive		R	S	G	R	T	R	R	L	Forward cacc atg agg tcc ggg cgc act cgg aga ctt tga<br>Reverse tca aag tct ccg agt gcg ccc gga cct

	Saccharomyces cerevisiae<br>Yeast	Serine/threonine-protein kinase HSL1p<br>Link	Tested<br>Positive	S	R	S	R	R	S	L	R	L	Forward cacc atg tca cgt tcg aga aga tcg cta cgc tta tga<br>Reverse tca taa gcg tag cga tct tct cga acg tga
	Trichophyton verrucosum<br>Yeast	Signal transduction protein Syg1<br>Link	Tested<br>Positive	F	R	T	R	R	T	R	R	L	Forward cacc atg ttt cgt act cgt cgt act cga cga tta tga<br>Reverse tca taa tcg tcg agt acg acg agt acg aaa
	Streptomyces sp.<br>Actinobacteria	Regulator protein<br>Link	Tested<br>Positive	A	R	C	R	Q	Y	R	R	L	Forward cacc atg gcc aga tgt cgc caa tac cga cga tta tga<br>Reverse tca taa tcg tcg gta ttg gcg aca tct ggc
	Myxococcus virescens<br>Proteobacterium	Hypothetical protein<br>Link	Tested<br>Positive	F	R	C	R	T	Y	R	R	L	Forward cacc atg ttc agg tgc agg acc tac agg agg ctg tgy<br>Reverse tca cag cct cct gta ggt cct gca cct gaa

	**Organism**	**Protein and Link**	**Test result**	**1**	**2**	**3**	**4**	**5**	**6**	**7**	**8**	**9**	**Primers for cloning 5'-3'**
**NON-STIMULATORY PEPTIDES**	*Streptococcus pyogenes*<br>Bacteria	M protein, serotype 6 and others<br>Link	Tested<br>Negative	S	R	K	G	L	R	R	D	L	Forward cacc atg tct cgt aaa ggt ttg cgt cgt gat ttg tga<br>Reverse tca caa atc acg acg caa acc ttt acg aga
	Streptococcus pyogenes<br>Bacteria	M protein, serotype 6 and others<br>Link	Tested<br>Negative	S	R	Q	G	L	R	R	D	L	Forward cacc atg tct cgt caa ggt ttg cgt cgt gat ttg tga<br>Reverse tca caa atc acg acg caa acc ttg acg aga
	Corynebacteria durum<br>Bacteria	ABC transporter substrate-binding protein<br>Link	Tested<br>Negative	S	R	T	R	R	T	R	R	M	Forward cacc atg tcc cgt acc cgt aga aca cgc cgc atg tga<br>Reverse tca cat gcg gcg tgt tct acg ggt acg gga
	Beta vulgaris subsp. vulgaris<br>Crop	OTU domain-containing protein At3g57810<br>Link	Tested<br>Negative	P	R	N	A	R	V	R	R	L	Forward cacc atg cct cgg aat gca aga gta aga aga ctg tga<br>Reverse tca cag tct tct tac tct tgc att ccg agg
	Mycobacterium tuberculosis<br>Bacteria	Transposase%2C degenerate<br>Link	Tested<br>Negative	H	R	P	R	R	C	L	R	L	Forward cacc atg cat cga ccc cgc cgc tgc cta cgc ctc tga<br>Reverse tca gag gcg tag gca gcg gcg ggg tcg atg
	Triticum aestivum<br>Crop	Abscisic acid-induced protein, partial<br>Link	Tested<br>Negative	R	R	P	A	R	R	R	R	L	Forward cacc atg cgg cga cca gca cga cgg cgc agg ttg tga<br>Reverse tca caa cct gcg ccg tcg tgc tgg tcg ccg
	Triticum aestivum<br>Crop	Abscisic acid-induced protein, partial<br>Link	Tested<br>Negative	R	R	P	E	R	R	R	R	L	Forward cacc atg cgg cga cca gaa cga cgg cgc agg ttg tga<br>Reverse tca caa cct gcg ccg tcg ttc tgg tcg ccg
	Triticum aestivum<br>Crop	Abscisic acid-induced protein, octamer<br>Link	Tested<br>Negative		R	P	E	R	R	R	R	L	Forward cacc atg cga cca gaa cga cgg cgc agg ttg tga<br>Reverse tca caa cct gcg ccg tcg ttc tgg tcg
	Triticum aestivum<br>Crop	Abscisic acid-induced protein, partial<br>Link	Tested<br>Negative	R	R	P	G	R	R	R	R	L	Forward cacc atg cgg cga cca gga cga cgg cgc agg ttg tga<br>Reverse tca caa cct gcg ccg tcg tcc tgg tcg ccg
	Triticum aestivum<br>Crop	Abscisic acid-induced protein, octamer<br>Link	Tested<br>Negative		R	P	G	R	R	R	R	L	Forward cacc atg cga cca gga cga cgg cgc agg ttg tga<br>Reverse tca caa cct gcg ccg tcg tcc tgg tcg
	Triticum aestivum<br>Crop	Fasciclin-like protein FLA19<br>Link	Tested<br>Negative	V	R	A	G	R	G	L	R	L	Forward cacc atg gtt cgg gca ggg cga ggg ctc cgc cta tga<br>Reverse tca tag gcg gag ccc tcg ccc tgc ccg aac
	Triticum aestivum<br>Crop	Fasciclin-like protein FLA19, octamer<br>Link	Tested<br>Negative		R	A	G	R	G	L	R	L	Forward cacc atg cgg gca ggg cga ggg ctc cgc cta tga<br>Reverse tca tag gcg gagccc tcg ccc tgc ccg
	Lactobacillus bifidum<br>Bacteria	Cardiolipin synthase<br>Link	Tested<br>Negative	F	R	S	Y	W	P	R	R	L	Forward cacc atg ttt cga agc tat tgg ccg cgt cgt ttg tga<br>Reverse tca caa acg acg cgg cca ata gct tcg aaa
	Turicella otitidis<br>Bacteria	NADPH-dependent ferric siderophore reductase<br>Link	Tested<br>Negative		R	W	Y	T	V	R	R	L	Forward cacc atg cgc tgg tac acc gtg cgc cgc ctc tga<br>Reverse tca gag gcg gcg cac ggt gta cca gcg
	Triticum aestivum<br>Crop	Unnamed Protein Product<br>Link	Tested<br>Negative	H	R	R	G	R	Q	R	R	L	Forward cacc atg cac cgc cgc ggc cgg cag cga aga ctt tga<br>Reverse tca aag tct tcg ctg ccg gcc gcg gcg gtg
	Triticum aestivum<br>Crop	Unnamed Protein Product<br>Link	Tested<br>Negative	G	R	R	E	R	R	R	R	L	Forward cacc atg ggc cgc agg gag cgg cgg cgg cgg ctg tga<br>Reverse tca cag ccg ccg ccg ccg ctc cct gcg gcc
	Triticum aestivum<br>Crop	Unnamed Protein Product<br>Link	Tested<br>Negative	D	R	P	R	R	R	R	R	L	Forward cacc atg gac cgg ccg cgc cga cgg cgg cgc ctg tga<br>Reverse tca cag gcg ccg ccg tcg gcg cgg ccg gtc
	Triticum aestivum<br>Crop	Ent-kaurene synthase<br>Link	Tested<br>Negative	F	R	S	N	R	R	L	R	L	Forward cacc atg ttc cgc tcg aac cgt cgt ctt cga ctc tga<br>Reverse tca gag tcg aag acg acg gtt cga gcg gaa
	Triticum aestivum<br>Crop	Ent-kaurene synthase, octamer<br>Link	Tested<br>Negative		R	S	N	R	R	L	R	L	Forward cacc atg cgc tcg aac cgt cgt ctt cga ctc tga<br>Reverse tca gag tcg aag acg acg gtt cga gcg
	Triticum aestivum<br>Crop	Unnamed Protein Product<br>Link	Tested<br>Negative	A	R	S	A	R	R	R	R	L	Forward cacc atg gcc cgt tcg gcg cga cgg cgt cgg ctg tga<br>Reverse tca cag ccg acg ccg tcg cgc cga acg ggc

Triticum aestivum<br>Crop	Triose-phosphate Transporter family domain cont. protein<br>Link	Tested<br>Negative	A	R	A	A	R	R	R	R	L	Forward cacc atg gcg cgc gcg gcg agg cgg agg cgg ctg tga<br>Reverse tca cag ccg cct ccg cct cgc cgc gcg cgc
Triticum aestivum<br>Crop	Unnamed Protein Product<br>Link	Tested<br>Negative	R	R	R	R	R	R	L	R	L	Forward cacc atg agg cgc cgc cgc cgg cgc ctc cgc ctc tga<br>Reverse tca gag gcg gag gcg ccg gcg gcg gcg cct
Triticum aestivum<br>Crop	Abscisic acid-induced protein, octamer<br>Link	Tested<br>Negative		R	P	V	R	R	R	R	L	Forward cacc atg cga cca gta cga cgg cgc agg ttg tga<br>Reverse tca caa cct gcg ccg tcg tac tgg tcg
Triticum aestivum<br>Crop	Triose-phosphate Transp. family domain cont. Protein, octamer<br>Link	Tested<br>Negative		R	A	A	R	R	R	R	L	Forward cacc atg cgc gcg gcg agg cgg agg cgg ctg tga<br>Reverse tca cag ccg cct ccg cct cgc cgc gcg
Streptococcus pyogenes<br>Bacteria	50S ribosomal protein L18<br>Link	Tested<br>Negative		R	Q	K	R	H	R	R	V	Forward cacc atg cgc caa aaa cgc cac cgt cgc gtc tga<br>Reverse tca gac gcg acg gtg gcg ttt ttg gcg
Mycobacterium bovis<br>Bacteria	Type I pantothenate kinase<br>Link	Tested<br>Negative		R	Q	W	R	A	L	R	M	Forward cacc atg agg cag tgg cgc gcg ctc cgt atg tga<br>Reverse tca cat acg gag cgc gcg cca ctg cct
Candida albicans<br>Yeast	1-Sterol 3-beta-glucosyltransferase<br>Link	Tested<br>Negative		R	A	S	R	G	S	R	L	Forward cacc atg aga gct tca cgg ggt tca aga ttg tga<br>Reverse tca caa tct tga acc ccg tga agc tct
Candida albicans<br>Yeast	Ceramide glucosyltransferase (EC 2.4.1.80) (GLCT-1)<br>Link	Tested<br>Negative		R	R	V	R	W	L	R	V	Forward cacc atg cga cga gtg cgg tgg ttg cgg gtg tga<br>Reverse tca cac ccg caa cca ccg cac tcg tcg
Candida albicans<br>Yeast	L-2-aminoadipate reductase large subunit<br>Link	Tested<br>Negative		R	E	Q	R	C	R	R	F	Forward cacc atg aga gaa caa aga tgt cgt aga ttc tga<br>Reverse tca gaa tct acg aca tct ttg ttc tct
Candida albicans<br>Yeast	Increased rDNA silencing protein 4.<br>Link	Tested<br>Negative		R	L	E	N	T	R	R	I	Forward cacc atg aga ctt gag aat acc cgg aga atc tga<br>Reverse tca gat tct ccg ggt att ctc aag tct
**Organism**	**Protein and Link**	**Test result**	**1**	**2**	**3**	**4**	**5**	**6**	**7**	**8**	**9**	**Primers for cloning 5'-3'**

Legend to Table S2

Green: positive stimulation of Vα3S1/Vβ13S1 TCR; red: no stimulation.

The HLA-C*06:02 anchors are labelled in yellow, the TCR contact residues in green.

Primer sequences are given for cloning in into pcDNA3.1D/V5-His-TOPO, as well as origin and protein name with link to sequence.

Peptides tested as octamers and nonamers are highlighted by colors.

Table S3. Redundant peptide epitopes with different origins and potential environmental exposure

Peptide sequence	Source protein	Expressed in	Accession
**YRSYRTRRM**	IQ domain-containing protein IQM3-like	*Nicotiana tabacum*: tobacco	Link
		Coffea eugenioides et Arabica: coffee	Link<br>Link
		Capsicum annuum (Spanish pepper)	Link
		Solanum tuberosum: potatoe	Link
	IQ domain-containing protein IQM3-like isoform X2	*Sesamum indicum*: Sesame	Link
**ARSFRSLRL**	uncharacterized protein LOC103417966	*Malus domesticus*: apple	Link
	DNA replication/repair protein RecF	*Pseudomonas* sp.	Link
